# A Personalized Haplotype‐Resolved Near‐Gapless Genome Framework for Somatic Variant Discovery in Hepatocellular Carcinoma

**DOI:** 10.1002/advs.76856

**Published:** 2026-07-28

**Authors:** Jiazheng Lin, Yao Xiao, Shuangzi Luo, Yifan Li, Wenfei Zhang, Xiaoyu Zhang, Xinyi Wang, Di Wu, Jieru Hong, Yongkun Luan, Xun Huang, Yixiu Wang, Ningbo Wu, Xiaoxiao Hu, Fei Zhao, Wei Li, Xiao Luo, Bi Shi

**Affiliations:** ^1^ Hunan Research Center of the Basic Discipline for Cell Signaling Hunan Provincial Key Laboratory of Medical Virology College of Biology Hunan University Changsha Hunan China; ^2^ Department of General Surgery Xiangya Hospital Central South University Changsha China; ^3^ National Clinical Research Center for Geriatric Disorders Xiangya Hospital Central South University Changsha China; ^4^ International Joint Research Center of Minimally Invasive Endoscopic Technology Equipment & Standards Changsha China; ^5^ Department of Neurosurgery Renmin Hospital of Wuhan University Wuhan China

**Keywords:** centromeric remodeling, haplotype‐resolved genome assembly, hepatocellular carcinoma, precision oncology, structural variants

## Abstract

Cancer genome analysis still relies largely on universal reference genomes, which can obscure patient‐specific variation in repetitive, polymorphic, and structurally complex regions. Here, we generated personalized, haplotype‐resolved near‐gapless assemblies from paired hepatocellular carcinoma (HCC) tumor and matched normal tissues using PacBio HiFi, ultra‐long Oxford Nanopore, and Hi‐C sequencing. The matched normal assembly served as a personalized reference for tumor analysis, improving read alignment relative to GRCh38, reducing multi‐mapping, and achieving alignment performance comparable to T2T‐CHM13. This patient‐specific coordinate framework identified additional somatic small variants and structural variants, including variants missed or ambiguously represented by standard references. These assemblies also improved access to centromeric regions that are poorly captured by standard references, identifying patient‐specific tumor‐associated changes in assembled centromeric sequence content and repeat composition. Haplotype‐resolved transcriptomic analysis identified 205 allelic pairs with allele‐specific expression, including six dual protein‐coding tumor‐restricted candidates. Multi‐omic integration further prioritized candidate regulatory alterations in *MET* and *HSD17B2*, supported by allele‐dependent luciferase reporter activity. These findings support a patient‐centric assembly framework that complements standard references for resolving coding and non‐coding somatic alterations in HCC and provides a foundation for future cohort‐scale validation.

## Graphical abstract

A patient‐specific, haplotype‐resolved genome framework improves detection and interpretation of somatic variants in hepatocellular carcinoma. Using multi‐platform sequencing, the personalized assembly resolves complex regions including centromeres and MHC/HLA loci, enhances structural variant discovery, and links regulatory alterations to allele‐specific expression and chromatin features, providing a foundation for precision analysis in clinical HCC genomes.

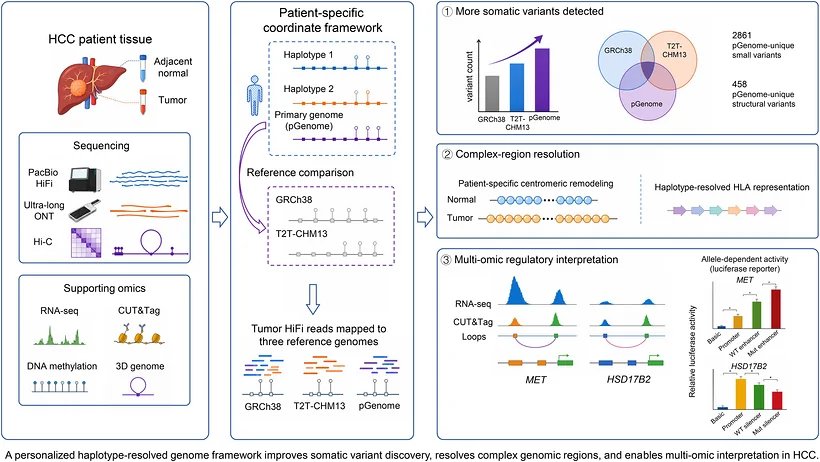

## 1 Introduction

Hepatocellular carcinoma (HCC), the third‐leading cause of cancer‐related deaths globally, arises from multifactorial interactions among environmental exposures, genetic susceptibility, and chronic liver disease progression [1]. Although targeted therapies and immune checkpoint inhibitors improve outcomes in subsets of patients, interindividual response heterogeneity stems in part from tumor molecular complexity [2]. This heterogeneity spans driver mutations, including *TP53* and *CTNNB1* alterations [3, 4], structural variations (SVs) that remodel chromosomes [5], and aneuploidy‐associated regulatory dysregulation [6]. Together, these features motivate patient‐specific genomic representations that can support precision oncology, although clinical implementation remains constrained by sample availability, sequencing resources, and the difficulty of analyzing structurally complex genomic regions.

A central obstacle is the mismatch between an individual tumor genome and the universal coordinate systems used for analysis. Most current workflows align reads to GRCh38, whose unresolved centromeric, telomeric, and other complex repeat regions can compromise interpretation in two ways: ambiguous alignment in repetitive regions can inflate false‐positive mutation calls, and reference‐specific coordinate differences can complicate the representation and interpretation of tumor‐specific SVs, including chromothripsis and viral integration events [7]. Although the Telomere‐to‐Telomere consortium released the gap‐free human genome assembly T2T‐CHM13 in 2022 [8], its haploid origin limits its ability to represent diploid variation and patient‐specific genome remodeling during carcinogenesis, including sequence diversity around integration sites and clonally amplified regions often observed in HCC [9]. Individual genomic diversity also introduces reference bias when a single reference genome is applied universally [10]. This bias is particularly important in cancer genomics, where *de novo* personalized assemblies may mitigate alignment artifacts and improve genomic profiling. Consistent with this concept, pioneering work by Xiao et al. using cultured normal and tumor cells derived from the same breast cancer donor demonstrated that a *de novo* assembled personalized genome could serve as an individualized reference for cancer somatic mutation discovery, improving read alignment and somatic SNV/SV detection relative to GRCh38 [11]. Garg et al. also advanced chromosome‐scale haplotype‐resolved reconstruction in cancer genomes [12]. Together, these studies established an important foundation for personalized and haplotype‐resolved cancer genome analysis. A remaining gap is therefore not whether personalized assemblies can improve cancer variant detection, but how matched patient‐specific tumor–normal assemblies can be implemented as a broader discovery framework in patient‐derived tumor tissue, particularly for direct assembly‐level SV discovery, structurally complex region analysis, and integration of somatic variation with multi‐omic regulatory features.

Long‐read sequencing and Hi‐C‐based scaffolding have made personalized cancer genome assembly feasible and have already supported matched tumor–normal assembly studies [11]. Building on this technical foundation, Hifiasm‐based assembly workflows can integrate PacBio HiFi reads, ultra‐long Oxford Nanopore Technologies (ONT) reads, and Hi‐C data to generate accurate, high‐contiguity, parent‐free haplotype‐resolved assemblies, supporting the reconstruction of long repetitive and structurally complex regions [13, 14, 15, 16]. These developments provide a technical route toward sample‐centric genome representations that can complement reference‐based workflows. Reference‐based alignment methods nevertheless remain widely used for SV detection [17], largely because they are computationally efficient and have lower sequencing‐depth requirements. Alignment‐based SV inference can be challenged by repetitive contexts and ambiguous breakpoints, whereas assembly‐based approaches can reconstruct local sequence context and variant structure, improving breakpoint resolution and interpretability in specific settings [18]. These strategies are therefore complementary, and context‐specific use can provide a more comprehensive characterization of structural variation.

Here, we integrated PacBio HiFi, ultra‐long ONT, and Hi‐C sequencing to generate haplotype‐resolved *de novo* assemblies of paired tumor and adjacent normal liver tissues from an HCC patient. The normal‐derived primary assembly served as the personalized genome reference (pGenome), while the tumor assemblies enabled tumor–normal assembly‐level comparison for assembly‐based somatic SV discovery. We systematically characterized single nucleotide variants (SNVs), insertions/deletions (indels), and SVs across GRCh38, T2T‐CHM13, and pGenome frameworks, evaluated alignment‐based and assembly‐based variant detection strategies, and extended the analysis to complex genomic regions, including centromeres and the MHC/HLA locus. We further integrated RNA‐seq, CUT&Tag, DNA methylation, and Hi‐C data to connect pGenome‐resolved somatic alterations with chromatin state and transcriptional output. As a single‐patient proof‐of‐concept, this study provides a practical framework for evaluating how matched patient‐specific tumor–normal assemblies can complement standard references in resolving coding and non‐coding somatic variation, structurally complex genomic regions, and candidate regulatory alterations relevant to cancer genomics.

## 2 Results

### 2.1 Study Design and Construction of Phased and Reference‐Grade Personalized Genome Assemblies From Multi‐Platform Sequencing Data

To establish a comprehensive genomic framework for HCC analysis, we first addressed the challenge of constructing a phased reference‐grade genome. Normal and tumor tissues collected during surgical resection from a male patient with HCC were analyzed through a hybrid assembly strategy, which systematically integrated PacBio HiFi reads, ultra‐long ONT reads, and Hi‐C data (Figure 1a). This workflow combined *de novo* genome assembly, quality evaluation, scaffolding, and haplotype phasing, using Hifiasm [16] for contig assembly and HapHiC [19] for chromosomal anchoring. The pipeline generated three assemblies for each sample: a primary assembly and two haplotype‐resolved assemblies. The primary normal assembly, designated pGenome, was selected as the reference for downstream analyses.

**FIGURE 1:**
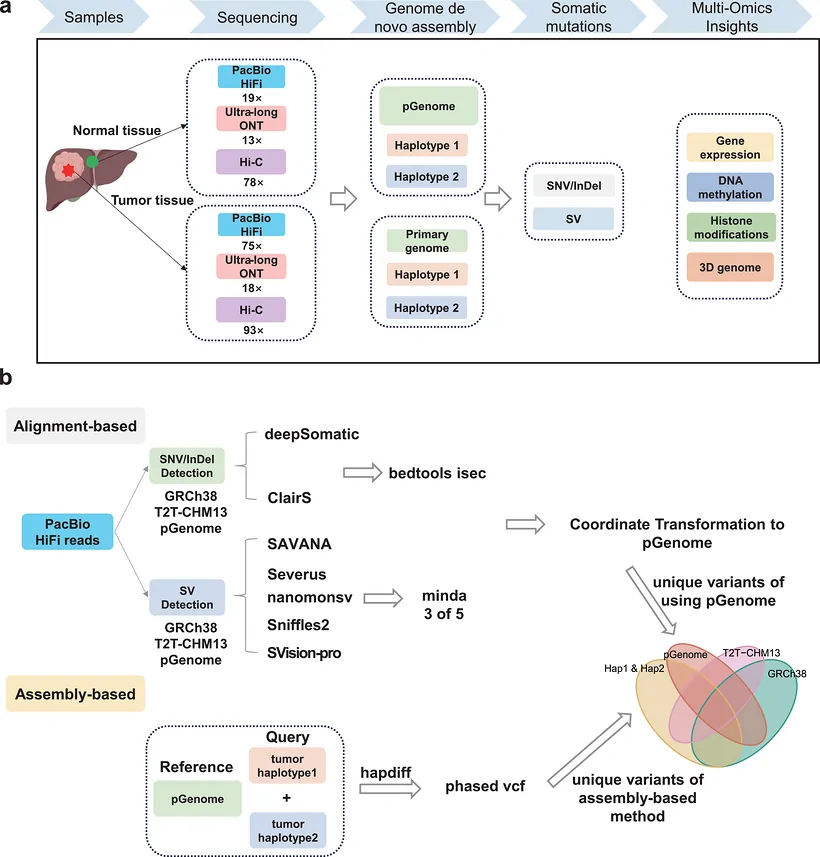
Overview of the study design and variant detection strategies. (a) Study design schematic. Whole‐genome sequencing data from paired normal and tumor tissues were generated using PacBio HiFi, Nanopore, and Illumina platforms. Hybrid de novo assembly pipelines integrated multi‐platform data to generate tissue‐specific diploid genome reconstructions, comprising a primary genome and two phased haplotypes (haplotype 1 and haplotype 2). The resulting personalized genome (pGenome) served as the reference for downstream somatic variant detection (SNVs, InDels, and SVs). Functional impacts of identified variants were systematically characterized through multi‐omics integration. (b) Variant detection strategies. (1) Alignment‐based method: PacBio HiFi reads were aligned against three reference genomes (GRCh38, T2T‐CHM13, and pGenome) to evaluate reference bias in variant calling. (2) Assembly‐based method: Tumor‐specific haplotypes were directly compared to pGenome to detect somatic structural variations.

The pGenome assembly spans 3,080,856,517 bp across autosomes and sex chromosomes and achieves telomeric completeness for 19 chromosomes (Figure 2a). Compared with GRCh38, pGenome has higher contig continuity (contig NG50: 83.85 Mb versus 56.41 Mb) and fewer unresolved gaps (313 versus 1,094). At the scaffold level, pGenome achieves a scaffold NG50 of 154.46 Mb, comparable to T2T‐CHM13 (154.26 Mb) and higher than GRCh38 (145.14 Mb) (Table 1). Functional annotation using Liftoff [20] successfully mapped 62,278 of 63,086 GENCODE v46 genes (98.72%), including 19,861 of 20,065 protein‐coding genes (98.98%), and 251,866 of 254,070 transcripts (99.13%).

**FIGURE 2:**
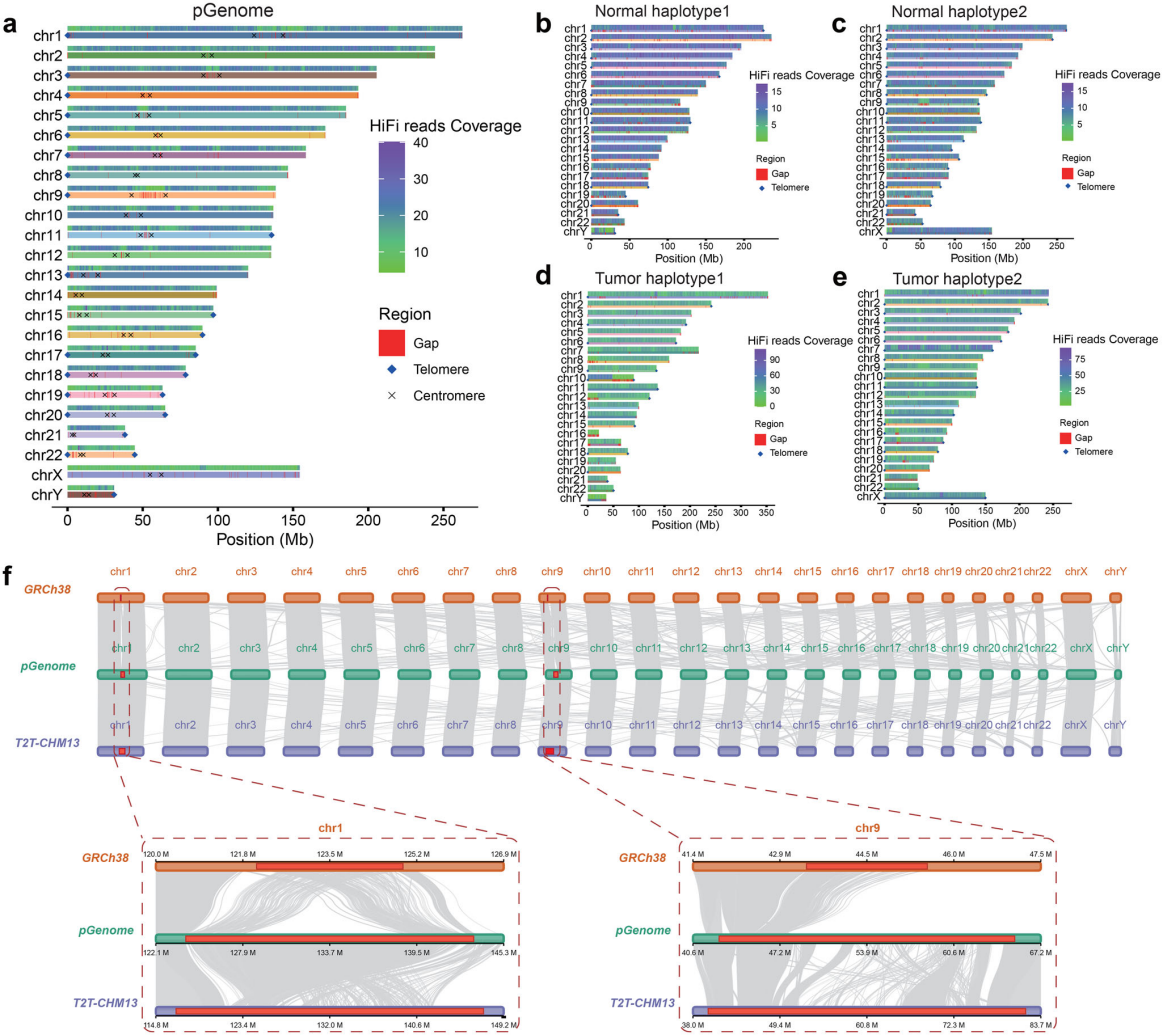
Genome assembly overview. (a–e) Assembly results for the following genomes: (a) Primary personalized genome (pGenome) constructed through hybrid assembly (PacBio HiFi + ultra‐long ONT + Hi‐C), the upper track displays HiFi read coverage across each chromosome, while the lower track indicates chromosomal length. Centromeric regions are demarcated between paired black multiplication signs (×), with red vertical lines representing assembly gaps. Blue diamonds at chromosomal termini denote telomeric sequences. (b) Normal haplotype 1 genome. (c) Normal haplotype 2 genome. (d) Tumor haplotype 1 genome. (e) Tumor haplotype 2 genome. (f) Genomic collinearity analysis among GRCh38, pGenome, and T2T‐CHM13. Representative centromeric regions on chr1 and chr9 are highlighted in the main panel and shown in enlarged inset views, with red blocks denoting centromeric regions.

**TABLE 1:** Comparative assembly statistics of GRCh38, T2T‐CHM13, and pGenome.

Summary	GRCh38	T2T‐CHM13	pGenome
Assembled bases (Gb)	2.92	3.05	3.09
Unplaced bases (Mb)	11.42	0	7.95
Gaps	1094	0	313
Number of contigs	949	24	457
Contig NG50 (Mb)	56.41	154.26	83.85
Number of scaffolds	194	24	68
Scaffold NG50 (Mb)	145.14	154.26	154.46

The primary and haplotype‐resolved assemblies showed variable but overall sufficient quality for downstream analyses, with contig N50, scaffold N50, BUSCO completeness, and QV metrics shown in Figure 2b–e and summarized in Table 2.

**TABLE 2:** Summary of quality assessments of assemblies.

Statistics	Normal			Tumor		
	pGenome	Haplotype1	Haplotype2	Primary genome	Haplotype1	Haplotype2
Assembled bases (Gb)	3.09	2.73	3.07	3.15	2.9	3.08
Unplaced bases (Mb)	7.95	32.91	14.46	2.74	5.71	5.63
Gaps	313	848	742	132	469	166
Number of contigs	457	1036	978	194	611	245
Contig N50 (Mb)	83.85	11.79	14.11	105.13	83.85	101.04
Number of scaffolds	68	120	103	50	61	43
Scaffold N50 (Mb)	154.46	139.44	154.46	149.72	172.71	149.72
QV	49.77	36.35	39.72	64.06	38.56	56
Completeness (%)	98.12	99.40	99.40	98.23	99.66	99.49
BUSCO (%)	95.70	85.90	96.10	96.50	87.10	96.50
Genome fraction (%) (compare to GRCh38)	97.60	88.58	96.94	98.00	87.67	97.20
Correctly assembled (%)	94.88	90.00	93.95	90.71	87.63	93.27
Erroneous (%)	0.42	1.42	1.12	0.43	4.99	2.33

Genomic collinearity analysis revealed strong conservation across most autosomes when pGenome was compared with GRCh38 and T2T‑CHM13. Expected divergence was observed in centromeric regions (e.g., chr1 and chr9) and in the incompletely assembled Y chromosome, reflecting known challenges associated with highly repetitive ampliconic sequences (Figure 2f; Figure S1a,b). Relative to GRCh38, pGenome revealed 21,358 SVs, including 10,876 deletions, 6,672 insertions, 128 inversions, 1,421 translocations, and 2,261 copy‐number changes. Relative to T2T‐CHM13, pGenome revealed 20,550 SVs, including 8,675 deletions, 8,641 insertions, 76 inversions, 806 translocations, and 2,352 copy‐number changes. Based on the merged union of SV‐affected reference intervals in pGenome coordinates, these variants affected 96.61 Mb (3.14%) and 98.01 Mb (3.18%) of the pGenome reference, respectively.

Alignment performance testing with PacBio HiFi reads showed that pGenome achieved the highest gap‐compressed identity (99.74%), representing a 1.25‐percentage‐point increase over GRCh38 (98.49%) and a slight improvement over T2T‐CHM13 (99.65%). Key improvements included a 10.2% increase in mean mapped read length (19,781.7 bp versus GRCh38's 17,942.6 bp) and substantially reduced multimapping rates (4.8% versus 13.1%). Alignment specificity metrics further indicated lower redundancy relative to GRCh38 (1.048 versus 1.151). Redundancy values were nearly identical between pGenome (1.048) and T2TCHM13 (1.049), reflecting comparable alignment specificity across the two reference genomes (Table S1).

Remapping normal and tumor reads to pGenome enhanced SNV, indel, and SV detection compared with GRCh38 and T2T‐CHM13. Integrating assembly based and alignment based approaches further improved variant discovery (Figure 1b), illustrating the practical advantage of using a personalized reference for resolving patient specific genomic variation.

### 2.2 Personalized Assemblies Suggest Patient‐Specific Somatic Centromeric Remodeling in Hepatocellular Carcinoma

Centromeric aberrations have been linked to genomic instability in cancer and are frequently associated with chromosomal instability and aneuploidy [21]. However, conventional alignment‐based analyses using the GRCh38 reference genome have limited resolution in centromeric regions because of extensive repetitive sequences and mapping ambiguity. To examine centromeric alterations in this patient, we performed *de novo* assembly of centromeric sequences from matched normal and tumor genomes. As a descriptive assembly‐based observation, the tumor assembly contained more assembled centromeric sequence than the matched normal assembly (191.46 Mb versus 160.45 Mb; +19.3%; Figure S2a). However, chromosome‐level centromere length differences did not reach statistical significance in a paired two‐sided Wilcoxon signed‐rank test (*P* = 0.114). Given the technical challenges of assembling highly repetitive centromeric regions, we interpret these tumor‐normal length differences cautiously. At the chromosome level, chr1 showed a tumor‐higher centromeric signal with concordant support from sequencing‐depth‐normalized k‐mer abundance ratios in both primary‐assembly and haplotype‐summed analyses (k = 51), with an assembled length comparable in scale to the ∼30.45 Mb chr1 centromere in T2T‐CHM13 (Figure S2e,f).

To characterize the repeat composition of the assembled centromeric sequences, we annotated repetitive elements within *de novo* assembled centromeric regions using RepeatMasker [22]. In this patient, the assembled tumor centromeric regions showed a higher annotated satellite proportion than the matched normal assembly (63.34% versus 60.03%), accompanied by a higher proportion of simple repeats (17.39% versus 12.42%; Figure S2b). Further stratification of satellite annotations showed that α‐satellite sequences, annotated as ALR/Alpha repeats, represented the major satellite subclass in these assembled centromeric regions, with a larger annotated α‐satellite length in the tumor assembly than in the matched normal assembly (92.5 Mb versus 72.9 Mb; Figure S2c). In contrast, HSATII sequences showed a more modest difference (17.1 Mb versus 13.4 Mb), whereas interspersed repeats, including LINE1 and Alu elements, represented a smaller fraction of assembled tumor centromeric sequences (11.04% versus 16.18%; Figure S2d).

In this patient, bp‐weighted tumor‐normal centromeric CNV profiles showed chromosome‐specific centromeric copy‐number heterogeneity, including chr1 gain in the tumor genome (Figure S2g). Cohort‐level analysis of TCGA‐LIHC also identified chr1 centromeric gain in a subset of tumors, providing CNV context for this patient‐specific finding (Figure S2h).

### 2.3 Germline Haplotype Diversity and Reference Representation Bias in the MHC Locus

Beyond centromeric structural alterations, we extended our analysis to another clinically important genomic hotspot, the major histocompatibility complex (MHC), a ∼5 Mb locus on chromosome 6 coding for HLA genes among 40 000 documented alleles (IMGT/HLA v3.60) [23], representing the most polymorphic region in the human genome. The hyperpolymorphism of the MHC directly supports immune regulation by enabling HLA genes to mediate antigen presentation to CD4+ and CD8+ T cells [24]. To assess potential tumor‐specific alterations within this locus, we first compared the tumor and matched normal assemblies directly. This analysis identified only limited somatic structural variation, including 4 deletions, 2 insertions, and 1 duplication, indicating that large‐scale somatic remodeling of the MHC was modest relative to that observed in centromeric regions.

We next examined how MHC architecture differs between pGenome and population references. Comparative analysis of the MHC region between GRCh38 and pGenome identified 82 structural variations, including 45 deletions, 31 insertions, 2 contractions, 2 duplications, and 2 inversions (Figure S3a). Extending this comparison to T2T‐CHM13, we observed 33 deletions, 28 insertions, 3 contractions, 2 duplications, and 1 inversion across the MHC locus (Figure S3b).

Within the MHC locus, we further assessed the organization of HLA‐related genes. While GRCh38 (GENCODE v46) annotates 19 HLA genes, T2T‐CHM13 contains an expanded representation of this locus, annotating 37 HLA genes. pGenome confirmed these genes (encoding 147 transcripts) within chr6:28,463,513–33,501,622 and identified additional structural variations, including a key inversion between *HLA‐DRB1* and *HLA‐DRB5* (Figure S3c). Given the critical role of HLA polymorphism in adaptive immune responses [25], we implemented a dual‐strategy approach: 1) haplotype‐resolved assemblies integrated with the IMGT/HLA database [23] via SpecHLA [26], and 2) reference‐independent, read‐based HLA typing using SpecImmune [27]. The assembly‐based method resolved ambiguous HLA calls (e.g., *HLA‐E/DQB2*; Figure S3d) and refined the discordant *HLA‐DRB1* assignment to HLA‐DRB1*09:59, which showed the highest sequence concordance with the normal haplotype 1 assembly (Table S2 and Figure S3e).

### 2.4 Benchmarking Somatic SNV and SV Detection on the COLO829 Truth Set Across three Reference Genomes

To assess somatic variant calling across reference frameworks using the COLO829 dataset, we benchmarked SNV and SV calling on GRCh38, T2T‐CHM13, and pGenome. Available COLO829 truth sets differ by variant class and coordinate framework and are not fully reference‐neutral, especially in repetitive or structurally complex regions. Therefore, benchmarking metrics were interpreted as framework‐specific comparative measures rather than absolute estimates of cross‐reference calling accuracy. We first assembled the COLO829BL sample, which resulted in a high‐quality assembly suitable for downstream analyses (Table S3). We then compared two coordinate harmonization strategies: VCF‐liftover and BAM‐liftover. In the VCF‐liftover approach, reads were independently aligned, and variants were called directly on each reference genome. In contrast, the BAM‐liftover strategy followed the approach described by Paulin et al. [28], where reads were aligned to T2T‐CHM13 or pGenome, and the resulting BAM files were lifted over to GRCh38 using levioSAM2 [29] before variant calling and evaluation were performed uniformly on GRCh38 (Figure 3a).

**FIGURE 3:**
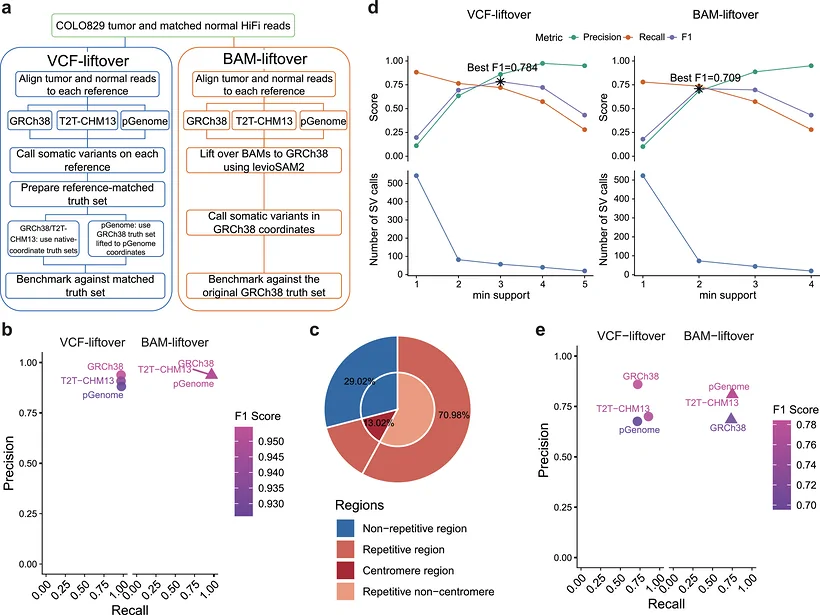
Benchmarking somatic SNV and SV detection on COLO829 across three reference genomes. (a) Workflow schematic of the VCF‐liftover and BAM‐liftover strategies for COLO829 benchmarking across GRCh38, T2T‐CHM13, and pGenome. (b) Somatic SNV benchmarking across the three genomes using VCF‐liftover and BAM‐liftover. (c) Annotation of apparent false‐positive SNVs, showing enrichment in repetitive and low‐mappability regions. (d) Performance of consensus SV calls using different numbers of callers. (e) Somatic SV benchmarking across the three genomes using VCF‐liftover and BAM‐liftover.

For SNV detection, we used both deepSomatic [30] and ClairS [31], and generated consensus calls. The COLO829 small‐variant truth set was obtained from Ha et al. [32]. Since their study provides a curated set of high‐confidence SNVs only, we restricted the small‐variant benchmarking analysis to SNVs. Under the BAM‐liftover strategy, SNV calling showed comparable precision, recall, and F1‐scores across GRCh38, T2T‐CHM13, and pGenome in this GRCh38‐anchored evaluation (Figure 3b and Table S4). However, under the VCF‐liftover strategy, using pGenome as the reference produced 4,779 apparent false positives against the GRCh38‐based truth set (Table S5). Among these, 70.98% were annotated in repetitive regions and 13.02% in centromeric regions, consistent with the previous observation that centromeric sequences exhibit substantial individual‐specific variation (Figure 3c). Read‐level validation using bam‐readcount [33] showed strong support for 4,763 of these sites, while 16 appeared to be germline variants, suggesting that many of these apparent false positives likely reflect incompleteness of the GRCh38‐based truth set rather than technical calling errors.

SV detection was also evaluated using both VCF‐liftover and BAM‐liftover. Five somatic SV callers were used: SAVANA [34], Severus [35], nanomonsv [36], Sniffles2 [37], and SVision‐pro [38] (Table S6). Consensus SVs were generated using minda [35], with caller‐support thresholds optimized on GRCh38 using the COLO829 truth set (obtained from Espejo Valle‐Inclan et al. [39]): ≥2 callers for BAM‐liftover SVs (excluding SAVANA, which was not compatible with BAM‐liftover outputs) and ≥3 callers for VCF‐liftover SVs, which yielded the highest F1‐scores (Figure 3d). Under the BAM‐liftover strategy, T2T‐CHM13 and pGenome showed higher apparent precision and recall than GRCh38 in this GRCh38‐anchored evaluation (Figure 3e and Table S7). In contrast, under the VCF‐liftover strategy, pGenome showed lower apparent precision and recall against the GRCh38‐based truth set (Figure 3e and Table S7). Inspection of the 23 SVs classified as apparent false positives under this benchmarking framework revealed that 6 overlapped centromeric regions and 10 overlapped highly repetitive regions. Notably, all 23 SVs were visually confirmed using SVhawkeye [40] (Figure S4a–c), indicating that these events likely represent real structural variants absent from the current GRCh38‐based COLO829 truth set, which incompletely captures variants in complex and repetitive genomic regions [39], rather than technical calling artifacts, consistent with observations reported by Paulin et al. [28].

### 2.5 Application of a Discovery‐Oriented Small‐Variant Detection Pipeline to Hepatocellular Carcinoma using Personalized Genomes

Because the primary goal of the HCC analysis was to identify somatic variants uniquely resolved in pGenome rather than to benchmark performance against a GRCh38‐based truth set, we used the VCF‐liftover strategy. Calls generated on GRCh38 and T2T‐CHM13 were subsequently lifted over to pGenome coordinates for cross‐reference comparison. Among 13,530 GRCh38‐based SNVs and 806 indels detected by both callers (Figure 4a), 14,242 small variants (99.3%) successfully mapped to pGenome (Figure 4b), with 13,142 variants overlapping with pGenome‐based calls (Figure 4c). This pattern was also observed for T2T‐CHM13‐based variants: of 13,742 T2T‐CHM13‐based SNVs and 812 indels (Figure 4a), 14,502 small variants (99.6%) mapped to pGenome (Figure 4b), with 13,388 overlapping variants (Figure 4c). Notably, pGenome‐based detection identified more small variants than either the GRCh38‐based or the T2T‐CHM13‐based approach. Comparison with aggregate somatic variant records from TCGA‐LIHC [41] and CLCA [4] showed that SNV records predominated in this patient and in both HCC cohorts. By contrast, annotation‐level distributions differed across datasets, likely reflecting differences in sequencing strategy, variant filtering, reference genome, and annotation pipeline (Figure S5a,b).

**FIGURE 4:**
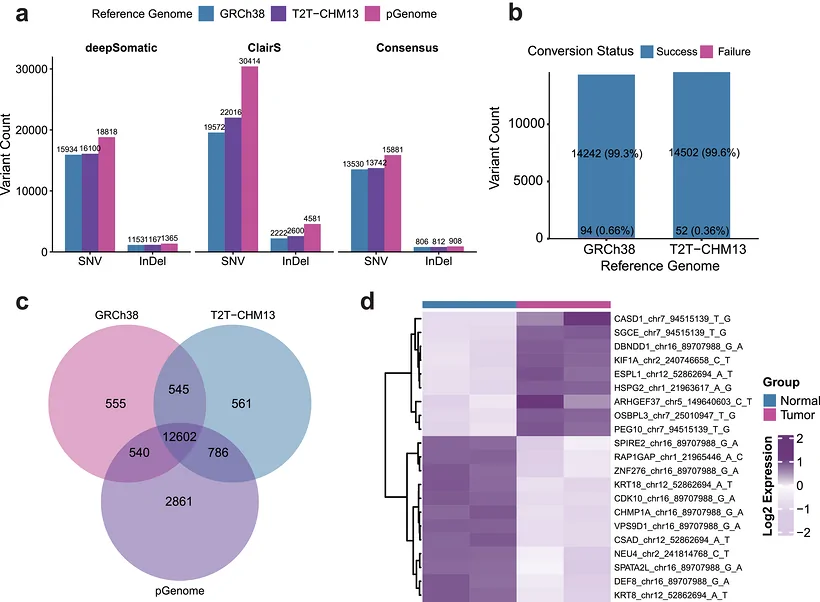
Somatic SNV/InDel detection using PacBio HiFi reads with GRCh38, T2T‐CHM13, and pGenome references. (a) The number of somatic SNVs/indels detected by deepSomatic and ClairS, as well as the overlap of variants identified by both tools. (b) Cross‐reference mapping success rates for somatic small‐variant coordinates originally identified using GRCh38 and T2T‐CHM13 when remapped to pGenome. (c) Venn diagram showing the overlap of variants detected in GRCh38, T2T‐CHM13, and pGenome references. (d) Heatmap visualization of RNA‐seq expression profiles for GTEx‐predicted pGenome‐unique SNV‐regulated genes. Color gradient reflects z‐score normalized expression levels.

pGenome‐based calling identified 15,881 SNVs and 908 indels (Figure 4a). Cross‐reference comparison further identified 2,861 pGenome‐unique variants (Figure 4c). Among these 2,861 pGenome‐unique variants, 2,733 were SNVs, of which 2,725 were supported as somatic by bam‐readcount [33], and 8 were consistent with possible germline origin. In total, 961 variants met the high‐confidence criteria (allele frequency > 0.5, supporting reads ≥ 10), whereas 979 variants were annotated as gene‐associated variants, including 358 high‐impact variants affecting 151 genes. To evaluate the potential functional relevance of these pGenome‐unique variants without overinterpreting pathway‐level signals, we next focused on gene‐level annotations and expression associations. For example, the pGenome‐unique *TMEM255B* variant (chr13: 119631295 C > T) was annotated as deleterious (SIFT [42] score: 0.05) and was associated with significantly reduced *TMEM255B* expression in tumor tissues (log2FC = −1.35, FDR < 0.05).

To further assess the potential regulatory effects of pGenome‐unique SNVs, which were detected in this sample only when using the pGenome reference and were not necessarily absent from public datasets, we converted their coordinates to the GRCh38 reference framework and queried precomputed significant cis‐eQTL variant‐gene associations from the GTEx database [43]. This analysis identified 35 pGenome‐unique SNVs that matched 123 significant GTEx cis‐eQTL variant‐gene associations, involving 87 gene symbols. Figure 4d shows the RNA‐seq expression pattern for the subset of these GTEx‐linked genes that were differentially expressed between tumor and normal samples in our dataset. Representative examples included cancer‐related genes such as *CDK10* [44] (chr16:89707988 G>A, nominal *P* = 2.14 × 10^−33^), *ULK1* [45] (chr12:131563683 G>A, nominal *P* = 9.98 × 10^−6^), *KIF1A* [46] (chr2:240746658 C>T, nominal *P* = 1.68 × 10^−5^) and *HSPG2* [47] (chr1:21963617 A>G, nominal *P* = 9.98 × 10^−5^).

To validate selected pGenome‐unique somatic SNVs, we focused on the 358 high‐impact pGenome‐unique small variants affecting 151 genes. We intersected these 151 genes with the OncoKB [48] Cancer Gene List and identified 12 cancer‐related genes for targeted validation. For each of these 12 genes, we selected one representative SNV for Sanger sequencing based on sufficient tumor read support and primer design feasibility. In total, 11 of the 12 prioritized SNVs (91.7%) were successfully confirmed, whereas the *AKT3* variant (chr1:257208896 A>T) could not be validated because PCR amplification failed (Figure S6 and Table S8).

### 2.6 Application of a Discovery‐Oriented SV Detection Strategy to Hepatocellular Carcinoma using Personalized Genomes

Using the same discovery‐oriented strategy, we next analyzed somatic SVs in HCC across the three reference genomes. Calls generated on GRCh38 and T2T‐CHM13 were subsequently lifted over to pGenome coordinates for cross‐reference comparison. Using a stringent multi‐caller consensus strategy (≥3 callers), pGenome identified 611 high‐confidence somatic SVs, compared with 207 using GRCh38 and 320 using T2T‐CHM13 (Figure 5a).

**FIGURE 5:**
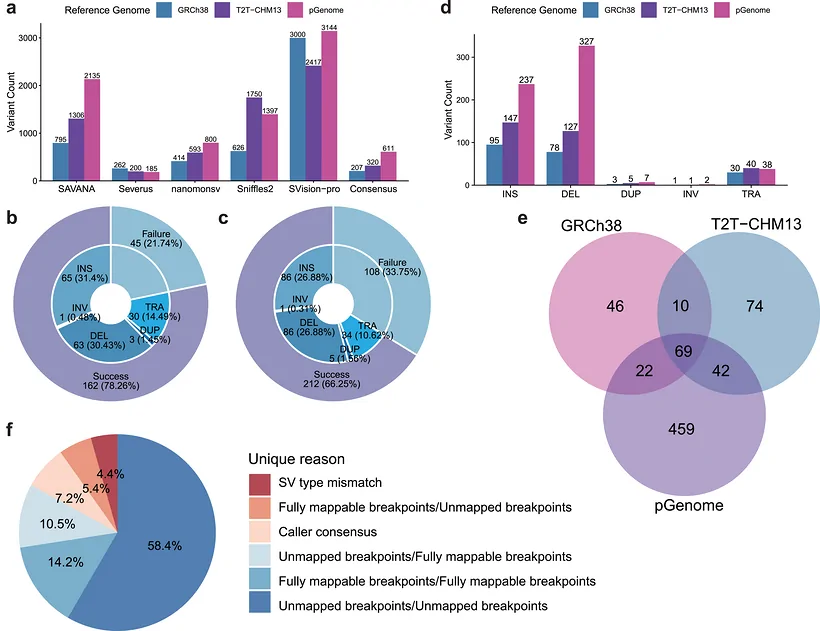
Somatic SV detection using PacBio HiFi reads with GRCh38, T2T‐CHM13, and pGenome references. (a) The number of somatic SVs detected by five different callers, along with the number of consensus variants supported by at least three callers. (b) The number of variant types detected using GRCh38, T2T‐CHM13, and pGenome as reference genomes. (c,d) Cross‐reference mapping success rates for somatic SV coordinates originally identified using (c) GRCh38 or (d) T2T‐CHM13 when liftovered to pGenome. (e) Venn diagram showing the overlap of SVs detected in GRCh38, T2T‐CHM13, and pGenome references. (f) Distribution of pGenome‑unique SV reasons. For breakpoint mappability, the left and right terms denote mapping status in GRCh38 and T2T‑CHM13, respectively.

Overall, pGenome identified a larger set of high‐confidence somatic SV candidates. Relative to GRCh38, pGenome detected 142 additional insertions, 249 deletions, 1 inversion, 4 duplications, and 8 translocations; relative to T2T‐CHM13, it identified 90 insertions, 200 deletions, 1 inversion, 2 duplications, and 2 fewer translocations (Figure 5b). Lift‐over analysis further revealed substantial reference‐specific differences, with only 78.26% (162/207) of GRCh38‐based SVs and 66.25% (212/320) of T2T‐CHM13‐based SVs successfully mapped onto pGenome coordinates (Figure 5c, d). Notably, pGenome identified 459 SVs that were not recovered in the GRCh38‐ or T2T‐CHM13‐based analyses (Figure 5e). Among these, 20.04% (92/459) overlapped gene bodies, including genes implicated in cancer‐related processes, such as *TERT*, *MUC2*, *PRKCA*, and *COL4A1*. Genomic annotation showed that pGenome‐unique SVs were highly enriched in challenging genomic regions, with 99 SVs located within centromeres and 291 overlapping repetitive elements. Validation with VaPoR [49] identified high‐confidence support for 158 of 287 evaluable SVs (VaPoR score > 0.15), while IGV inspection confirmed clear evidence for 10 of 12 unevaluable translocation events, collectively supporting the reliability of a subset of pGenome‐unique SVs.

To investigate why these SVs were missed by GRCh38 or T2T‐CHM13, we classified pGenome‐unique SVs by SV type consistency, caller consensus, and breakpoint mappability. Using the unique mapping status of ±2 kb breakpoint‐flanking sequences in both references, SVs were grouped into six categories. We found that 58.4% of SVs were unmappable in both references, indicating missing or highly complex regions, while 10.9% were mappable to only one reference. Additionally, 5.4% of SVs resulted from SV type mismatches and 7.2% failed multi‐caller consensus filtering (Figure 5f).

To support the reliability of pGenome‐unique SVs, four representative events from distinct reference‐dependent categories were selected for orthogonal validation. These included Minda_178, an SV type mismatch detected as an insertion in pGenome but interpreted as a deletion in GRCh38; Minda_248 and Minda_93, representing unmapped/fully mappable and unmapped/unmapped breakpoint patterns, respectively; and Minda_176, a complex breakpoint‐mappability case in which breakpoint‐flanking sequences aligned to multiple distant loci in GRCh38 and T2T‐CHM13 but were resolved in pGenome. PCR amplification and Sanger sequencing confirmed the predicted breakpoint junctions of these representative SVs (Figure S7a–d).

Other representative cases further illustrated how reference choice affected SV interpretation. Minda_278 was detected as a deletion in pGenome but interpreted as an insertion in T2T‐CHM13, similar to the reference‐dependent SV type inversion observed for Minda_178 (Figures S8b and S10a). Caller consensus–related unique SVs primarily resulted from differences in caller support. Minda_268 met the consensus criterion in pGenome with support from SAVANA, nanomonsv, and SVision‐pro but failed in GRCh38 due to insufficient caller support (Figures S8c and S9b). Similarly, Minda_63 did not pass the consensus threshold in T2T‐CHM13, where it was supported by only two callers (Figures S8d and S10b).

Structural differences between references affected SV interpretation even when breakpoints were fully mappable. In Minda_102, breakpoint‐flanking sequences mapped to GL000225.1 in GRCh38 and to chr21 in T2T‐CHM13, leading to cross‐chromosomal mappings and inconsistent SV detection across assemblies (Figure S8e). Minda_172 was detected only against pGenome because a 2,889‐bp TE‐rich allele present in GRCh38 and T2T‐CHM13 was absent from pGenome, causing the allele to appear as a relative insertion (Figures S8g and S9d and S10c). Minda_22 represents a fully mappable/unmapped breakpoint case, in which breakpoint flanking sequences mapped uniquely to GRCh38 but lacked corresponding locations in T2T‐CHM13; notably, ±2 kb flanking sequences aligned to two distant loci in GRCh38, disrupting SV detection (Figures S8f and S9c). Conversely, Minda_202 exemplifies an unmapped/fully mappable event, with flanking sequences mapping uniquely to T2T‐CHM13 but absent from GRCh38, suggesting incomplete representation in GRCh38 (Figures S8h and S10d). Although clear SV signals were visible in tumor HiFi read alignments to T2T‐CHM13, no caller detected the event, likely due to its centromeric location. This SV was ultimately resolved to the centromeric region of chr19 in pGenome, highlighting the utility of personalized genomes for resolving SVs in highly complex regions.

### 2.7 Comparison of Assembly‐Based and Alignment‐Based Methods for Somatic SV Detection

While alignment‐based approaches are widely used for SV detection, some large‐scale or complex rearrangements are difficult to resolve using alignment signals alone [17]. Assembly‐based approaches provide an alternative by enabling haplotype‐aware characterization via genome sequence comparison. To visualize and systematically assess assembly‐derived SVs, we first applied syRI [50] to compare the tumor haplotype‐resolved assemblies against pGenome, which revealed large‐scale chromosomal rearrangements, including a ∼90 Mb duplication on chromosome 1 in haplotype 1 and a haplotype‐specific translocation between chromosomes 16 and 8 (Figure 6a).

**FIGURE 6:**
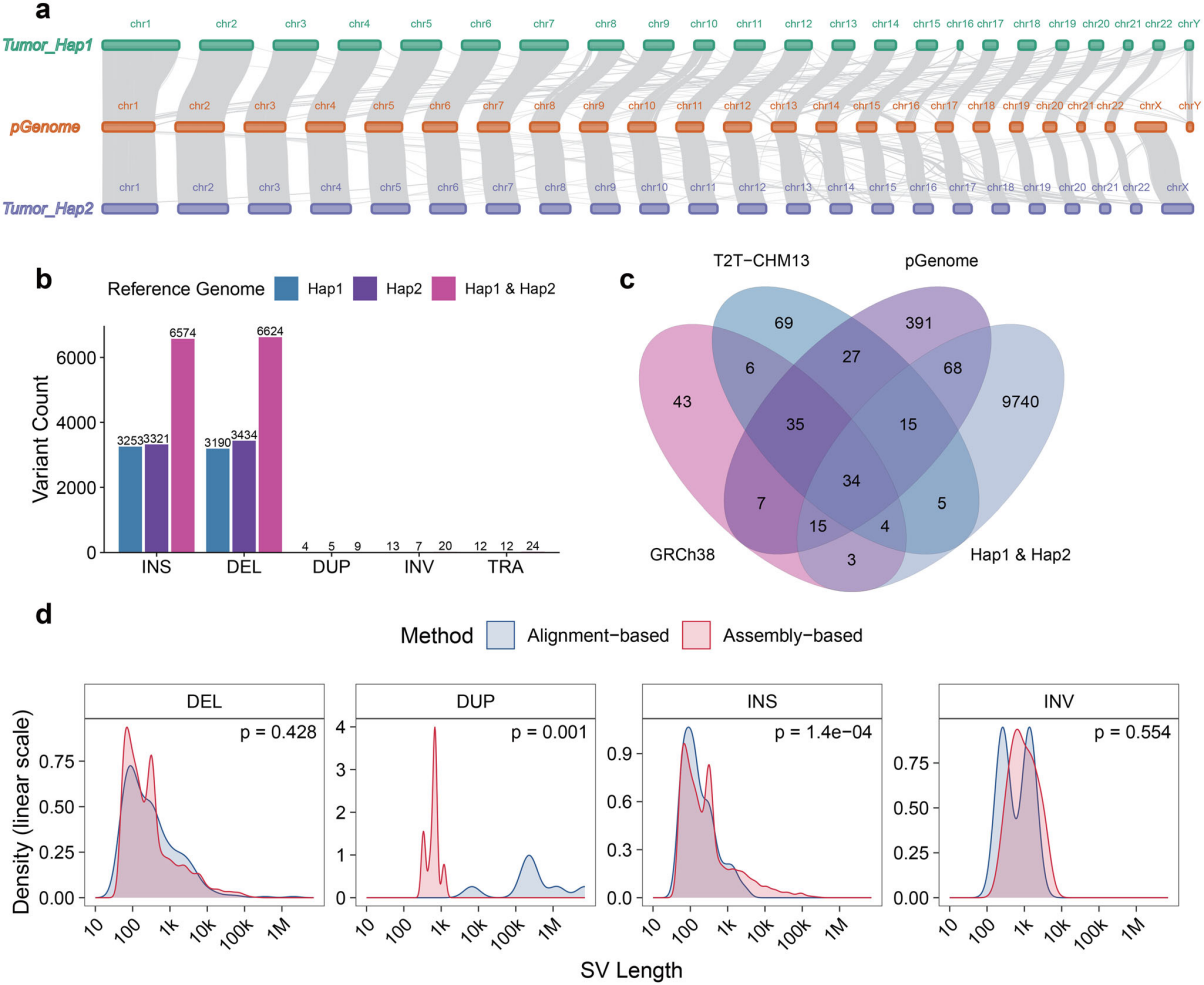
Structural variant (SV) detection using alignment‐based and assembly‐based approaches. (a) Genomic collinearity analysis of pGenome, tumor haplotype 1 genome, and tumor haplotype 2 genome. (b) Counts of structural variant types detected in tumor haplotype 1, tumor haplotype 2, and the merged haplotype SV set derived from assembly‐based analysis. (c) Venn diagram showing the overlap of SVs detected by the alignment‐based method (using GRCh38, T2T‐CHM13, and pGenome references) and the assembly‐based method. (d) Length distributions of structural variants detected by alignment‐based and assembly‐based approaches.

Hapdiff [51] identified 12,923 non‐redundant tumor SV events after collapsing haplotype‐level calls, including 12,595 heterozygous and 328 homozygous events. Because homozygous events are present on both haplotypes, these corresponded to 13,251 haplotype‐level SV alleles assigned to haplotypes 1 and 2 (6,472 and 6,779), including 6,624 deletions, 6,574 insertions, 24 BND/translocation events, 20 inversions, and 9 tandem duplications (Figure 6b). To evaluate complementarity between strategies, we compared assembly‐based SVs with alignment‐based SV calls. After harmonizing variant representations, 9,740 SVs were found to be uniquely detected by the assembly‐based approach and absent from the alignment‐based callset (Figure 6c). Assembly‐based SVs were enriched for larger events, particularly ∼1 kb insertions (*P* = 1.4 × 10^−^^4^) (Figure 6d). The quality of the hapdiff SV callset was further evaluated using TT‐Mars [52]. TT‐Mars classified 9,896 SVs as true positives, 729 as false positives, and 2,298 SVs as not assessable, yielding a high proportion of validated events among evaluable SVs. Notably, the assembly‐specific SV in *RAB31* (chr18:9815883–9816736 DEL) was identified as a splice acceptor variant with high impact by SnpEff [53] and was associated with significantly reduced *RAB31* expression in tumor tissues (log2FC = −1.28, FDR < 0.05). Functional annotation showed that 4,649 assembly‐specific SVs overlapped 2,964 genes. KEGG enrichment analysis of these genes identified several pathways that remained significant after Benjamini–Hochberg correction, including MAPK signaling, calcium signaling, focal adhesion, and platelet activation (FDR < 0.05; Figure S11), with representative affected genes such as *RAP1A*, *ATP2B4*, and *AKT3*.

### 2.8 Tumor‐Restricted Allelic Expression of Differentially Expressed Genes

To dissect allele‐specific regulatory patterns in cancer, we conducted haplotype‐resolved transcriptome analysis on matched tumor‐normal samples. Unlike conventional analyses based on a single haploid reference genome, this strategy enabled the detection of 8,374 allelic pairs through BLASTn [54] and MCScanX [55] analyses. Of these, 2,557 (30.5%) showed measurable expression (Figure S12a), with 682 pairs exhibiting significant allelic divergence (|log2FC| > 1 and FDR < 0.05). Tumor samples contained 205 ASE‐positive allelic pairs, including 6 dual protein‐coding pairs that showed significant allelic bias in tumor but did not meet the same ASE threshold in matched normal tissue; these pairs were therefore defined as tumor‐restricted ASE candidates (Figure S12b). Representative examples included *FCGR2A* (hap1‐dominant, log2FC = −1.23, FDR = 0.011), a key modulator of immunosuppressive microenvironments [56] and *NCLN* (hap2‐dominant, log2FC = 1.32, FDR = 0.02), which is involved in multichannel membrane protein translocation to the endoplasmic reticulum and embryonic development [57].

### 2.9 Three‐Dimensional Genome Remodeling Associated With Somatic Variants

To examine three‐dimensional genome features associated with method‐specific variants, we performed Hi‐C‐based compartment analysis at 50‐kb resolution. Regions harboring variants uniquely detected by both methods showed a higher proportion of the transcriptionally active A compartment in tumor samples than in matched normal samples (alignment‐based: tumor 56.8% versus normal 49.6%; assembly‐based: tumor 50.7% versus normal 44.7%) (Figure 7a). We next quantified compartment transitions between normal and tumor within these bins. In both variant sets, B‐to‐A transitions were more frequent than A‐to‐B transitions (alignment‐based: B‐to‐A 12.98% versus A‐to‐B 4.16%; assembly‐based: B‐to‐A 9.84% versus A‐to‐B 4.37%) (Figure 7b), indicating that somatic variant‐overlapping regions were preferentially associated with compartment activation in the tumor sample.

**FIGURE 7:**
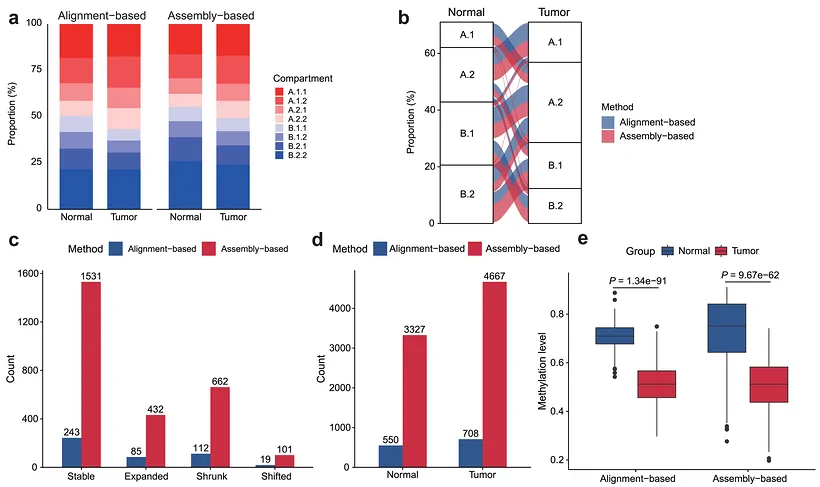
Analysis of unique mutation regions detected by alignment‐based and assembly‐based methods in compartments, TADs, loops, and DNA methylation levels in normal and tumor tissues (using pGenome as the reference genome). (a) Proportion of A/B compartments in unique mutation regions detected by the two methods. A/B compartments were further subdivided into multiple subtypes (e.g., A.1/A.2 and B.1/B.2 subclasses). (b) Transition between A/B compartments in unique mutation regions detected by both methods (A/B compartments were subdivided into A.1/A.2 and B.1/B.2 subclasses). (c) Changes in TADs associated with unique mutation regions detected by both methods. TAD overlap is defined as the size of the overlapping region between normal and tumor. Stable refers to overlap/normal > 0.9 & overlap/tumor > 0.9, Expanded refers to overlap/normal > 0.9 & overlap/tumor < 0.9, Shrunk refers to overlap/normal < 0.9 & overlap/tumor > 0.9, and Shifted refers to overlap/normal < 0.9 & overlap/tumor < 0.9. (d) The number of loops in TADs containing unique mutations detected by both methods. (e) DNA methylation levels in the regions of unique mutations detected by both methods.

Building on these observations, we next examined topologically associating domains (TADs) at 50‐kb resolution in these variant regions and observed consistent structural reorganization patterns across both detection methods. We classified tumor–normal differences as stable, expanded, shrunk, or shifted domains based on changes in boundary positions and domain span. The alignment‐based method detected 85 expanded, 112 shrunk, and 19 shifted TADs in tumors, whereas the assembly‐based method identified larger numbers of altered domains (432 expanded, 662 shrunk, and 101 shifted) (Figure 7c). Both methods identified increased chromatin loop formation at 10‐kb resolution in tumors compared with normal samples (Figure 7d). Variant‐associated regions also showed reduced DNA methylation (Figure 7e), consistent with a more transcriptionally permissive chromatin state in the tumor sample [58].

### 2.10 pGenome‐Centered Multi‐Omic Integration Prioritizes Candidate Regulatory Alterations

To explore whether pGenome‐resolved somatic variants were associated with regulatory changes in this HCC case, we integrated SNVs/SVs with transcriptomic, chromatin, DNA methylation, and Hi‐C profiles. Using the pGenome framework, which combined alignment‐based and assembly‐based variant sets, we identified 594 genes harboring SNVs or SVs in promoter, enhancer, or silencer regions. Among these genes, 194 exhibited significant differential expression (|log2FC| > 1, FDR < 0.05), with 126 upregulated and 68 downregulated genes (Figure 8a). Epigenetic profiling showed concordant chromatin‐state patterns: upregulated genes showed increased H3K4me3/H3K27ac signals along with reduced H3K27me3 modification at promoters, whereas downregulated genes displayed opposite chromatin patterns (Figure 8b).

**FIGURE 8:**
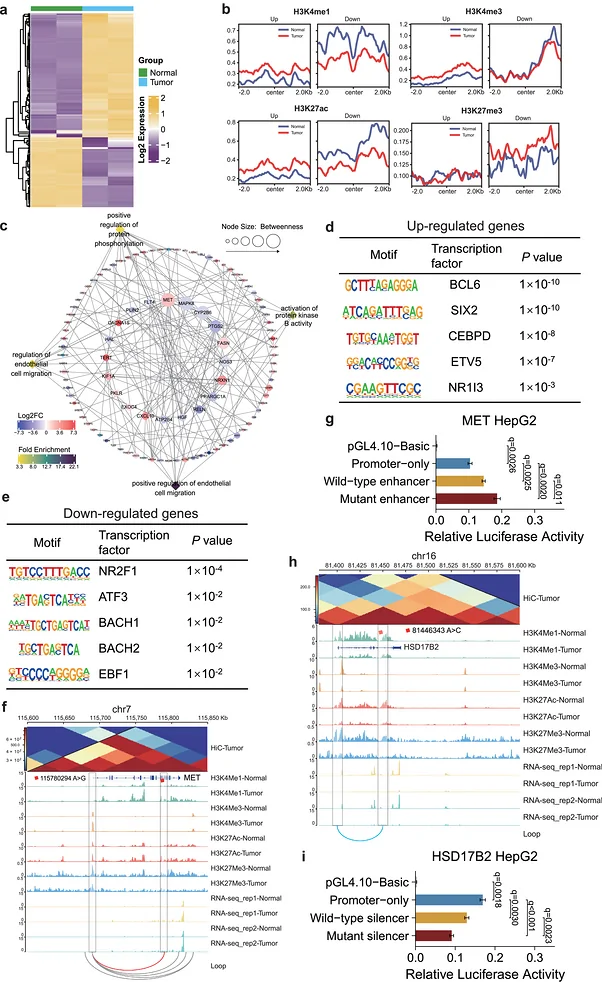
Genome‐wide epigenetic profiling of somatic variants and regulatory impacts. (a) Differentially expressed genes (DEGs) with somatic variants in regulatory regions. (b) Distribution of histone modifications (H3K4me1, H3K4me3, H3K27ac, H3K27me3) across up‐regulated and down‐regulated genomic regions. (c) Protein–protein interaction network and enriched biological processes for DEGs harboring somatic variants in regulatory regions. Rectangles denote GO‐enriched biological processes retained after Benjamini–Hochberg correction, with fold enrichment shown; circles represent genes. d, Motif enrichment analysis for upregulated DEGs with somatic variants in regulatory regions. (e) Motif enrichment analysis for downregulated DEGs with somatic variants in regulatory regions. (f) Integrated view of *MET* gene regulation: histone modifications (H3K4me1, H3K4me3, H3K27ac, H3K27me3), RNA expression, and 3D genome looping. Red diamonds indicate somatic SNV loci. (g) Luciferase reporter assay of the *MET* wild‐type and mutant regulatory alleles in HepG2 cells. (h) Regulatory landscape of *HSD17B2* gene, including histone modifications, RNA expression, and chromatin architecture. Red diamonds mark SNV positions. (i) Luciferase reporter assay of the *HSD17B2* wild‐type and variant‐containing regulatory fragments in HepG2 cells. For all luciferase reporter assays, *Firefly* activity was normalized to *Renilla*, and pairwise Welch‐test *P* values were adjusted by Benjamini–Hochberg FDR correction.

Functional enrichment analysis of the 194 differentially expressed variant‐associated genes revealed significant enrichment of biological processes related to endothelial cell migration and AKT/protein phosphorylation signaling, including positive regulation of endothelial cell migration (FDR = 0.0219, fold enrichment = 6.73) and activation of protein kinase B activity (FDR = 0.0365, fold enrichment = 22.10) (Figure 8c). Protein–protein interaction network analysis further identified the top 20 hub genes, including *MET* and *MAPK8*, based on betweenness centrality using the CytoNCA algorithm [59] (Figure 8c). Motif dissection implicated *BCL6*/*SIX2* and *NR2F1*/*ATF3* as candidate regulators of upregulated and downregulated genes, respectively (Figure 8d,e). We identified 20 oncogenes and 16 tumor suppressor genes (TSGs) carrying regulatory‐region mutations, including 9 significantly upregulated oncogenes such as *MET* (log2FC = 1.97, FDR = 3.21 × 10^−110^) and *TERT*, as well as dysregulated TSGs such as upregulated *CDKN2B* (log2FC = 1.87, FDR = 7.97 × 10^−5^) and downregulated *HSD17B2* (log2FC = −3.68, FDR = 1.04 × 10^−87^). Sanger sequencing confirmed the MET‐ and HSD17B2‐associated candidate regulatory SNVs in paired tumor and matched normal DNA (Figure S13a,b). To assess the regulatory activity of these candidate variants, we performed dual‐luciferase reporter assays using wild‐type and variant‐containing regulatory fragments amplified with primers listed in Table S9. For the A>G variant (chr7:115780294) within the *MET* super‐enhancer (Figure 8f), the mutant enhancer (G allele) significantly increased luciferase activity compared with the wild‐type enhancer in HepG2 cells (FDR = 0.011; Figure 8g), with the same direction of effect observed in 293T and Huh7 cells (Figure S14a and Table S11), indicating enhanced enhancer‐driven reporter activity. Normalization with *Renilla* luciferase controlled for transfection efficiency and well‐to‐well variation. This result was consistent with chromatin interaction data showing stronger promoter‐enhancer contacts involving the G allele, supporting a potential association between this variant‐containing enhancer and increased *MET* regulatory activity. Given the established role of *MET* signaling in HCC, this candidate regulatory alteration may be relevant to MET‐related oncogenic signaling, including MAPK/ERK and PI3K/AKT pathways [60]. At the *HSD17B2* locus, a tumor‐specific promoter‐silencer loop disruption coincided with an SNV (chr16:81,446,344 A>C; Figure 8h). In HepG2 cells, the variant‐containing silencer fragment significantly reduced luciferase activity compared with the wild‐type sequence (FDR = 0.0023; Figure 8i), with the same direction of effect observed in 293T and Huh7 cells (Figure S14b and Table S11), suggesting altered regulatory activity of this silencer fragment and its potential association with reduced *HSD17B2* expression. These reporter assays support allele‐dependent regulatory activity in an episomal reporter context but do not establish endogenous causal regulation or tumor‐driving function. Beyond point variants, structural alterations were associated with distinct epigenetic regulatory changes: The 71,947‐bp heterozygous promoter deletion at *CDKN2B* (chr9:22,081,255–22,153,202) on haplotype 1 was associated with increased *CDKN2B* expression, accompanied by increased H3K4me3/H3K27ac signals, reduced H3K27me3 signals (Figure S14c), and promoter hypomethylation (Figure S14d). In TCGA‐LIHC, higher *CDKN2B* expression was correlated with poorer prognosis (HR = 1.9, *P* = 7 × 10^−4^; Figure S14e). In contrast, the 72‐bp promoter deletion at *RNF213* (chr17:82,231,044–82,231,116, a pGenome‐unique variant; Figure S14f) in the putative HCC driver gene [61] overlapped a region with reduced H3K27me3 signals and was associated with increased *RNF213* expression (FC = 1.59, FDR = 9.04 × 10^−26^).

## 3 Discussion

This study uses a deeply profiled hepatocellular carcinoma (HCC) case to examine how haplotype‐resolved personalized assemblies can complement population references in cancer genome analysis. Population‐scale diploid and haplotype‐resolved assemblies have established the importance of representing germline structural diversity and reducing reference bias, while standard references such as GRCh38 and T2T‐CHM13 remain indispensable coordinate systems for cancer genomics [62]. Cancer analysis, however, adds a distinct somatic dimension: tumor genomes must be interpreted relative to the patient's own germline background, and somatic SVs, repeat‐associated remodeling, allele‐specific expression, and regulatory alterations may depend on patient‐specific sequence context. Previous work using cultured normal and tumor cells derived from the same breast cancer donor demonstrated that an individualized genome assembly can improve read alignment and somatic SNV/SV detection relative to GRCh38 [11]. Building on this foundation, the present study applies a matched tumor–normal dual‐assembly strategy to primary HCC tissue. The normal‐derived pGenome provides an individualized coordinate framework, whereas the tumor assemblies enable direct tumor–normal assembly‐level comparison. This design extends personalized genome analysis from reference‐guided somatic variant discovery to assembly‐based SV discovery, patient‐specific centromeric analysis, MHC/HLA haplotype representation, and pGenome‐centered integration of somatic variants with allele‐specific expression, chromatin state, three‐dimensional genome organization, and candidate regulatory activity. Thus, the contribution of this study lies in the primary‐HCC implementation of a tumor–normal dual‐assembly framework that links somatic variant discovery with complex‐region resolution and multi‐omic regulatory interpretation.

The pGenome framework improved the interpretability of somatic variation in genomic contexts where conventional reference‐based analyses are constrained by mappability, unresolved sequence or population divergence. Relative to GRCh38, pGenome provides higher continuity and fewer unresolved regions, while achieving alignment performance comparable to T2T‐CHM13. In small‐variant and structural‐variant analyses, pGenome recovered additional candidate somatic variants that were missed or ambiguously represented when reads were analyzed against GRCh38 or T2T‐CHM13. These findings support the practical value of personalized references for discovery‐oriented analyses, but they also show that benchmarking and coordinate‐conversion strategies should be selected according to the analytical objective. BAM‐liftover is best suited for GRCh38‐anchored benchmarking and annotation, whereas VCF‐liftover or direct per‐reference analysis is more appropriate when the goal is to preserve patient‐specific sequence context; these use cases are summarized in Table S10. Apparent false positives in a GRCh38‐based benchmark should therefore be interpreted cautiously, because some may reflect incompleteness of the truth set in complex regions rather than technical calling errors.

A major advantage of patient‐specific assembly is the ability to interrogate regions that are usually underrepresented in cancer genome studies. Centromeres are particularly difficult to study using standard alignment‐based workflows because their repeat‐rich architecture complicates read mapping and variant representation [63]. Recent haplotype‐resolved assemblies have shown that human centromeres vary extensively in α‐satellite array length and organization, highlighting why patient‐specific assemblies are needed to study these regions [64]. In this patient, the tumor assembly contained more assembled centromeric sequence than the matched normal assembly, accompanied by higher annotated satellite and simple‐repeat proportions within assembled centromeric regions. Repeat annotation further showed a larger annotated α‐satellite representation within the assembled tumor centromeric sequences. However, we interpret these observations cautiously. Chromosome‐level centromere length differences did not reach statistical significance, and only chr1 showed concordant support in both assembly‐based and k‐mer abundance analyses. Thus, the data support patient‐specific centromeric remodeling, rather than a general claim that α‐satellite expansion is a recurrent feature of HCC. Cohort‐scale long‐read studies will be required to determine whether similar centromeric alterations recur across patients and whether they are associated with chromosomal instability, clinical features or tumor evolution.

The MHC/HLA analysis illustrates a distinct, primarily germline, dimension of personalized assembly. Unlike the tumor‐normal centromere comparison, which addresses somatic remodeling, the MHC analysis focuses on haplotype‐resolved representation of a highly polymorphic immune locus within this patient. By combining assembly‐based HLA resolution with read‐based typing, we obtained allele‐resolved calls and identified structural differences across the MHC region that are difficult to interpret with a single reference representation, consistent with recent near‐complete genome analyses showing extensive complex variation across the MHC locus [64]. These results highlight the potential of personalized assemblies to improve analysis of immune‐relevant loci, while clinical applications such as immunotherapy stratification, transplantation matching or neoantigen interpretation will require prospective validation in larger and more diverse cohorts.

Beyond variant discovery, the personalized assembly provided a coordinate framework for linking somatic variants to transcriptional and epigenomic features. Haplotype‐resolved transcriptomic analysis identified tumor‐restricted ASE candidates, and pGenome‐centered multi‐omic integration prioritized candidate regulatory alterations at loci including *MET* and *HSD17B2*. Reporter assays supported allele‐dependent regulatory activity for selected variants, strengthening their prioritization. However, these assays were performed in an episomal reporter context and do not establish endogenous causal regulation or tumor‐driving function. Thus, the multi‐omic analyses should be interpreted as a variant‐prioritization framework rather than a complete mechanistic model of hepatocarcinogenesis.

From a translational perspective, this workflow should be regarded as a high‐resolution discovery framework rather than a routine clinical assay. Cultured tumor cells can be advantageous for genome sequencing and assembly because they provide relatively homogeneous, expandable tumor‐cell material with reduced stromal or normal‐cell admixture. However, cultured models and primary surgical specimens address different questions. Cell lines may undergo culture‐associated genetic or transcriptional evolution and clonal selection [65], whereas primary tumor tissues more directly reflect the sample complexity encountered in clinical cancer genomics, including tumor purity variation, normal‐cell admixture, stromal components, and intratumoral heterogeneity [2]. In this respect, the value of the present design is not that primary tissue is technically superior to cultured cells, but that it tests a patient‐specific assembly framework in a clinically realistic and technically challenging HCC specimen. PacBio HiFi WGS is the core data type for personalized assembly‐based somatic variant discovery, whereas Hi‐C supports chromosome‐scale scaffolding, haplotype phasing and assembly curation. Ultra‐long ONT sequencing, RNA‐seq and CUT&Tag can add repeat resolution or functional interpretation, but are not all essential for a reduced implementation. Even a reduced tumor‐normal HiFi WGS workflow with recommended Hi‐C support would currently remain in the several‐thousand‐US‐dollar range per patient for sequencing and library preparation alone, excluding personnel, computation, storage, interpretation, reporting, validation and optional assays. The current turnaround time is expected to be weeks rather than days, making this approach most suitable at present for selected research or translational settings, such as genomically complex, recurrent, drug‐resistant or otherwise unresolved tumors.

Several limitations remain. First, this is a single‐patient proof‐of‐concept, so the biological observations reported here should be interpreted as patient‐specific and hypothesis‐generating. Second, personalized assemblies complement rather than replace conventional reference‐based analyses, established cancer‐gene models and orthogonal validation. Third, assembly‐based approaches preferentially capture clonal or high‐abundance events, whereas subclonal SVs may be under‐represented when heterogeneous tumor variation is collapsed into haplotype assemblies. Fourth, assembly quality varied across haplotypes and samples, which may affect locus‐specific haplotype interpretation, particularly in repetitive or structurally complex regions. Finally, experimental validation covered only a subset of variants. Future cohort‐scale studies that combine long‐read personalized assemblies with standardized benchmarking, orthogonal validation, and clinical annotation will be needed to define when patient‐centric assembly provides the greatest benefit for cancer genome interpretation.

## 4 Conclusion

In summary, this study illustrates how matched tumor–normal haplotype‐resolved personalized genome assemblies can complement existing population references by supporting more resolved characterization of somatic variation, structural complexity, and regulatory remodeling in a primary cancer genome. The main value of this work is not the generation of a personalized assembly alone, but the use of a normal‐derived pGenome together with tumor assemblies as a patient‐specific framework for assembly‐level SV discovery, complex‐region interpretation, and multi‐omic regulatory prioritization. Although limited by its single‐patient design, this work provides a practical framework for future cohort‐scale studies that integrate phased genome assemblies with multi‐omic profiling. As long‐read sequencing technologies continue to improve in accuracy and accessibility, patient‐centric assemblies may become increasingly practical for selected precision oncology applications that require faithful reconstruction of structurally complex and regulatory genomic regions.

## 5 Experimental Section

### 5.1 Sample Collection

Paired specimens comprising HCC tumor tissue and adjacent non‐tumorous liver parenchyma were surgically resected from a treatment‐naïve HCC patient at Xiangya Hospital, Central South University. Immediately following surgical resection, all tissue specimens were snap‐frozen in liquid nitrogen and subsequently stored at −80°C to preserve biomolecular integrity for downstream analyses. The patient's blood test results were as follows: HBsAg‐negative, HBsAb‐positive, HBeAb‐negative, HBeAg‐negative, HBcAb‐negative, and HBcAb IgM‐negative. Sample input amounts and quality metrics for genomic, transcriptomic, and epigenomic experiments are summarized in Table S12.

### 5.2 Ethical Approval

This study complied with the “Ministry of Science and Technology (MOST) Guidelines on the Review and Approval of Human Genetic Resources” and has received official clearance for the export of human genetic materials and data from China. The study was also approved by the institutional review board of Xiangya Hospital (202308003‐2), and informed consent was obtained from the patient enrolled in this research.

### 5.3 RNA‐seq Sequencing and Data Analysis

Total RNA was extracted from normal liver and tumor tissues using the MolPure Cell/Tissue Total RNA Kit (Yeasen Biotechnology), with genomic DNA removed by in‐column RNase‐Free DNase treatment. Poly(A)‐enriched RNA libraries were prepared using the NEBNext Ultra RNA Library Prep Kit for Illumina (New England Biolabs) and sequenced on the Illumina NovaSeq X‐Plus platform, yielding 30–50 million 150‐bp paired‐end reads per library.

Raw reads were preprocessed with fastp (v0.23.4) [66] to remove adapters and low‐quality bases. High‐quality reads were aligned to the pGenome (the personalized primary genome assembly from normal tissue) using STAR (v2.7.11b) [67], and gene expression levels were quantified with HTSeq (v2.0.3) [68]. Differential gene expression analysis was performed using DESeq2 (v1.42.0) [69] with stringent thresholds (|log2 (fold change, FC)| > 1, FDR < 0.05). Enriched KEGG pathways were identified via ClusterProfiler (v4.10.0) [70].

### 5.4 CUT&Tag Sequencing and Data Analysis

CUT&Tag assays were performed on frozen tissue‐derived nuclei using the Hyperactive Universal CUT&Tag Kit (Vazyme Biotech). Nuclei were incubated with primary antibodies targeting H3K4me1 (ActiveMotif, #39635), H3K4me3 (#39060), H3K27ac (#39034), and H3K27me3 (#39155), followed by secondary antibody conjugation and tagmentation using pA‐Tn5 transposase. Libraries were amplified with 2×CAM (Vazyme Biotech) and sequenced on the Illumina NovaSeq X‐Plus platform (50–80 million 150‐bp paired‐end reads per library).

After adapter trimming and quality filtering with fastp (v0.23.4) [66], reads were aligned to pGenome using Bowtie2 (v2.5.4) [71]. Duplicates and low‐quality alignments were removed via deeptools’ alignmentSieve (v3.5.5) [72]. Peaks for histone modifications were called using MACS3 (v3.0.2) [73] with default parameters. Enhancer regions were annotated using the ROSE algorithm [74]. Visualization was performed using pyGenomeTracks (v3.9) [75].

### 5.5 Hi‐C Sequencing and Data Analysis

Cross‐linked nuclei (10^6^ cells) were digested with MboI (150 U), subjected to proximity ligation with T4 DNA ligase, and sheared to ∼400 bp. Biotinylated ligation junctions were enriched using streptavidin beads (Thermo Fisher), and libraries were prepared with the NEBNext Ultra II DNA Library Prep Kit. Sequencing was performed on the Illumina NovaSeq X‐Plus platform, generating ∼1 billion 150‐bp paired‐end reads per library.

Hi‐C reads were processed with fastp (v0.23.4) [66], aligned to pGenome using BWA (v0.7.18) [76], and analyzed for chromatin compartments (CALDER2, v0.7 [77]), topologically associating domains (TADs; HiCExplorer v3.7.6 [78]), and chromatin loops (Mustache v1.0.1 [79]).

### 5.6 PacBio HiFi Sequencing and Data Analysis

High‐molecular‐weight DNA was extracted using the CTAB method, purified with the QIAGEN Genomic‐tip Kit, and sheared using g‐TUBEs (Covaris). SMRTbell libraries were constructed with the SMRTbell Prep Kit 3.0 (PacBio) and sequenced on the PacBio Revio platform. Raw HiFi reads were mapped to the pGenome reference using pbmm2 (https://github.com/PacificBiosciences/pbmm2 v1.16) with default parameters.

Alignment quality metrics were derived from the resulting BAM files. Gapcompressed identity was calculated by pbmm2 as:

$$\begin{array}{ccc} & & 100\times \mathrm{matches}/\left(\mathrm{matches}+\mathrm{mismatches}\right.\\ & & \;\left.+\;\mathrm{insertion}\;\mathrm{events}+\mathrm{deletion}\;\mathrm{events}\right) \end{array}$$

where consecutive indels are counted as a single event. Multimapping rate was defined as: (secondary reads + supplementary reads)/total reads, and alignment redundancy as: total reads/primary reads.

### 5.7 Nanopore Ultra‐Long Sequencing and Data Analysis

DNA was extracted using the MagAttract HMW DNA Kit (Qiagen), and libraries were prepared on the PromethION P48 platform (ONT R10.4 flow cells). Sequencing generated pod5 files, which were filtered (Q score ≥7) and converted to bam format using Dorado (v0.7.0).

### 5.8 DNA Methylation Analysis

High‐quality HiFi reads were generated using CCS (https://github.com/PacificBiosciences/ccs v6.4) with the “–hifi‐kinetics” option to extract base kinetics information and produce consensus sequences containing kinetic tags. 5mC methylation at CpG motifs was predicted using Jasmine (https://github.com/pacificbiosciences/jasmine v2.4.0), which added base modification (“MM”) and modification probability (“ML”) tags to the reads. The reads were then aligned to pGenome using pbmm2 (https://github.com/PacificBiosciences/pbmm2 v1.16) with default high‐accuracy parameters, preserving kinetic signals for downstream analysis. Methylation sites and probabilities were quantified using the pileup‐based script aligned_bam_to_cpg_scores.py from pb‐CpG‐tools (https://github.com/PacificBiosciences/pb‐CpG‐tools v2.3.2), which analyzed CpG‐specific kinetic signals in the aligned reads.

### 5.9 Genome Assembly Pipeline

Haplotype‐resolved genome assemblies were constructed for both tumor‐adjacent normal tissue and tumor tissue samples using Hifiasm (v0.19.9‐r616) [16]. The assembly strategy integrated PacBio HiFi reads, ultra‐long ONT reads, and Hi‐C data, generating three contig sets: primary.fa, haplotype1.fa, and haplotype2.fa. Contigs were filtered to remove mitochondrial DNA contamination by alignment against the T2T‐CHM13 ChrM.fa reference using BLAST (v2.16.0+) [54] with stringent parameters (‐evalue 1e‐5 ‐perc_identity 0.8 ‐max_target_seqs 5). Contigs with >80% sequence similarity to mitochondrial sequences were discarded. Chromosome scaffolding was performed using HapHiC (v1.0.6) [19], followed by manual curation with Juicebox (v2.15) [80] to optimize chromosomal structure. Gap closure was implemented through a three‐stage process: ONT‐based gap filling with TGS‐GapCloser (v1.2.1) [81] using ultra‐long ONT reads; secondary refinement with PacBio HiFi reads; and final gap resolution using NextDenovo (v2.5.2) [82]‐assembled contigs. For haplotype‐resolved assemblies, haplotype‐specific reads identified with Canu (v2.2) [83] SplitHaplotype were incorporated during gap closure.

The resulting assemblies were designated as pGenome (the personalized primary genome assembly from normal tissue) and haplotype1/haplotype2 (haplotype‐resolved diploid assemblies).

### 5.10 Assembly Evaluation

Assembly continuity for the normal, tumor, and haplotype‐resolved assemblies was summarized using contig N50 and scaffold N50, calculated with assembly‐stats (v17.02; https://github.com/rjchallis/assembly‐stats). For cross‐assembly comparisons among GRCh38, T2T‐CHM13, and pGenome, we report contig NG50 and scaffold NG50 as normalized continuity metrics for assemblies with different total lengths. Genome completeness was assessed with BUSCO (v5.6.1) [84] by analyzing the presence of conserved single‐copy orthologs from the appropriate lineage dataset, reporting percentages of complete, fragmented, and missing genes. To validate assembly accuracy, raw HiFi reads were aligned to the genome using pbmm2 (v1.16) with default parameters, and the resulting mapping rate was calculated. Merqury (v1.3) [85] was employed to assess k‐mer completeness and assembly consistency, generating a QV (quality value) score derived from k‐mer spectra analysis. QUAST (v5.2.0) [86] was used to quantify genome‐wide assembly metrics including total assembly size and GC content. Structural validation was performed using Flagger (v1.1.0) [87], which detects different types of mis‐assemblies, correctly assembled regions, and structural errors across the genome.

### 5.11 Genome Annotation

The assembled genome was annotated using Liftoff (v1.6.3) [20] to transfer structural annotations from the GRCh38 reference genome (GENCODE release v46), with high‐quality alignments between the reference and assembled genomes used to maximize annotation accuracy.

### 5.12 Centromere Identification

The centromeric regions were identified using Centromics (https://github.com/ShuaiNIEgithub/Centromics v0.3), and repeat sequences were annotated using RepeatMasker (v4.1.7) [22].

### 5.13 Motif Enrichment Analysis

Motif enrichment analysis was performed using HOMER (v5.1) [88].

### 5.14 SNV and Indel Detection

SNVs and indels were identified by aligning tumor and matched tumor‐adjacent normal HiFi reads to three reference genomes (GRCh38, T2T‐CHM13, and pGenome) using pbmm2 (https://github.com/PacificBiosciences/pbmm2 v1.16) with default parameters (–log‐level INFO –sort). Variant calling was performed independently using ClairS (v4.0.0) [31] and deepSomatic (v1.6.1) [30] in paired tumor‐normal mode. To ensure high‐confidence somatic variants, results from both callers were intersected using bcftools (v1.17) [89] with strict filtering criteria (‐i ‘FILTER = “PASS”’), retaining only concordantly identified variants. The final high‐confidence SNVs and indels were defined as variants supported by both ClairS and deepSomatic within each reference‐genome analysis. Variant annotation was performed using SnpEff (v5.2) [53].

### 5.15 Structural Variant Detection

Alignment‐based SV calling was performed using five somatic callers: SAVANA (v1.3.5) [34], Severus (v1.6) [35], nanomonsv (v0.8.0) [36], Sniffles2 (v2.2) [37], SVision‐pro (v2.5) [38]. The outputs were integrated using minda [35] with parameters: –min_support = 3 –min_size 50 –tolerance 500, generating a high‐confidence consensus SV set. Assembly‐based SV calling was performed using SyRI (v1.7.0) [50] and hapdiff (v0.9) [51]. Genomic collinearity analysis was visualized using NGenomeSyn (v1.41) [90]. Variant annotation was performed using SnpEff (v5.2) [53].

### 5.16 Genome Coordinate Conversion

To enable variant mapping between T2T‐CHM13/GRCh38 and pGenome reference assemblies, coordinate conversion was performed through a multi‐step alignment pipeline. First, whole‐genome alignments were generated using minimap2 (v2.27‐r1193) [91] with default parameters, producing pairwise alignment format (PAF) records. These alignments were subsequently converted to UCSC chain format using transanno (https://github.com/informationsea/transanno v0.4.5). CrossMap (v0.6.3) [92] was then employed to convert variant coordinates in both VCF files and genomic region annotations between assemblies. For read‐level coordinate conversion, alignment files (BAM) generated against T2T‐CHM13 or pGenome were lifted over to GRCh38 coordinates using levioSAM2 [29].

### 5.17 Computational Validation of Somatic SNVs and SVs

Somatic SNVs were validated at the read level using bam‐readcount (v1.0.1) [33] to quantify allele‐specific read support in tumor and matched normal BAM files. Alignment‐based somatic SVs were validated using VaPoR [49] with default parameters; SVs with a VaPoR score > 0.15 were considered supported. Assembly‐based somatic SVs were validated using TT‐Mars (v1.2) [52] by comparing SV representations against haplotype‐resolved assemblies. All somatic SV calls were visually inspected using SVhawkeye [40] to confirm breakpoint consistency and read‐level support.

### 5.18 Benchmarking using the COLO829 Dataset

PacBio HiFi reads for COLO829 and its matched normal COLO829BL were obtained from https://downloads.pacbcloud.com/public/revio/2023Q2/COLO829. Somatic SV detection under VCF‐liftover and BAM‐liftover strategies was evaluated using minda [35]. Somatic SNVs were benchmarked using bcftools [89] by comparing detected variants against the curated COLO829 truth set.

### 5.19 Allele Identification and Haplotype Comparison

To identify allele‐specific genes and compare haplotypes, the longest transcripts from Haplotype 1 (Hap1) and Haplotype 2 (Hap2) were aligned using BLASTn (v. 2.16.0+) [54]. MCScanX (version 1.0.0) [55] was then applied to define collinear synteny blocks between Hap1 and Hap2. Within these blocks, gene pairs exhibiting high sequence similarity (identity >97%) were classified as reliable allele pairs (A and B).

For allele‐specific expression (ASE) analysis, RNA‐seq reads from normal and tumor tissues were mapped to the Hap1 and Hap2 reference sequences separately using HISAT2 (v. 2.2.1) [93]. Gene‐level read counts were quantified with HTSeq (v. 2.0.3) [68], and differential expression analysis was performed using DESeq2 (v. 1.42.0) [69]. To ensure robust ASE identification, genes with zero counts across all samples in both haplotypes were excluded from downstream analysis. Hap1‐ and Hap2‐derived read counts were compared separately in normal and tumor tissues, and allelic gene pairs with significant haplotype‐biased expression (|log2FC| > 1 and FDR < 0.05) were classified as ASE‐positive. Allelic pairs that were ASE‐positive in tumor but not in matched normal tissue were operationally defined as tumor‐restricted ASE candidates, whereas pairs significant in both tissues were considered shared ASE.

### 5.20 PCR Validation using Sanger Sequencing

DNA samples from normal liver tissue and tumor tissue were extracted using the MolPure Cell/Tissue DNA Extraction Kit (Yeasen Biotech Co., Ltd, Shanghai, China). A total of 12 SNVs with variant allele frequencies (VAFs) ranging from 0.5 to 1, together with 4 SVs, were selected from candidate SNVs and SVs discovered by pGenome‐based and cross‐reference analyses. Primers flanking the specific point mutations were designed using Primer3. Mutation‐flanking regions were amplified from both control and tumor DNA templates using Phanta Super‐Fidelity DNA Polymerase (Vazyme Biotech Co., Ltd, Nanjing, China). The PCR conditions included an initial denaturation at 94°C for 30 s, followed by 35 cycles of denaturation at 94°C for 10 s, annealing at 60°C for 20 s, and extension at 72°C for 30 s. The PCR products were then gel‐purified using the MolPure Gel Extraction Kit (Yeasen), cloned into a TA Cloning Kit (Vazyme), and sequenced at Sangon Biotech (Shanghai, China).

### 5.21 Dual‐Luciferase Reporter Assay

Promoter sequences of approximately 1.2 kb were cloned into the pGL4.10 firefly luciferase reporter vector (Promega, USA). Candidate regulatory fragments, including paired wild‐type and tumor/variant‐containing fragments, were PCR‐amplified and inserted immediately upstream of the corresponding promoter sequence. The empty pGL4.10‐Basic vector was included to assess background reporter activity, whereas the promoter‐only construct was included for each locus as the baseline reporter construct. All plasmids were confirmed by Sanger sequencing. Reporter assays were conducted in 293T, HepG2, and Huh7 cells cultured in DMEM (Biochannel, China) supplemented with 10% FBS (Biochannel, China) at 37°C with 5% CO_2_. Cells were seeded in 24‐well plates 24 h before transfection. Each well was transfected with 2 µL Lipofectamine 3000 reagent (Thermo Fisher Scientific, USA), 1000–1200 ng firefly reporter plasmid, and 100 ng pGL4.73‐Renilla plasmid (Promega, USA) as an internal control. Cells were lysed 24 h after transfection, and firefly and Renilla luciferase activities were measured using the Dual‐Luciferase Reporter Assay Kit (Vazyme, China) on a BioTek Synergy H1 microplate reader (Agilent Technologies, USA). Firefly activity was normalized to Renilla activity for each well, and the resulting Firefly/Renilla ratios were further normalized to the corresponding promoter‐only construct within each cell line and experiment. Percent changes were calculated relative to the paired wild‐type fragment.

## Author Contributions

The study was designed by B.S., X.L., and W.L. Data analysis was performed by J.L., Y.W., B.S., and X.L. Y.X. collected the sample and supervised the third‐generation sequencing. S.L. and D.W. conducted CUT&Tag and Hi‐C experiments under the supervision of W.L. Y. Li, W.Z., X.Z., and X.W. performed PCR validation of SNVs and SVs. Y. Li and Xun H. cloned the promoter and enhancer fragments and inserted them into plasmids. X. Hu, Y. Luan, F.Z., N.W., and J.H. contributed to experimental methods and technical support. The manuscript was drafted by J.L., W.L., X.L., and B.S., with critical revisions from all authors.

## Conflicts of Interest

The authors declare no conflicts of interest.

## Clinical Trial Registration

Not applicable. This study was not a clinical trial.

## Supporting information




**Supporting File**: advs76856‐sup‐0001‐SuppMat.docx.

## Data Availability

The accession number for all raw sequencing data reported in this paper in the Genome Sequence Archive (GSA) for Human is HRA010100.
