# Supplementary material for: A Personalized Haplotype‐Resolved Near‐Gapless Genome Framework for Somatic Variant Discovery in Hepatocellular Carcinoma
Source: Adv Sci (Weinh). 2026 Jul 28:e76856. Online ahead of print. doi: 10.1002/advs.76856 (PMC13410810; doi:10.1002/advs.76856)
Supplement: Supplementary file 1 — Supporting File: advs76856‐sup‐0001‐SuppMat.docx. [file ADVS-9999-e76856-s001.docx]

**Supplementary Tables and Figures**

**Supplementary Tables**

**Supplementary Table 1.** Performance metrics of PacBio HiFi read alignment to GRCh38, T2T-CHM13, and pGenome.

| Summary | GRCh38 | T2T-CHM13 | pGenome |
| --- | --- | --- | --- |
| Mean gap-compressed sequence identity (%) | 98.49 | 99.65 | 99.74 |
| Mean mapped read length (bp) | 17,942.6 | 19,771.3 | 19,781.7 |
| Mapped bases (Gb) | 57.72 | 57.97 | 57.97 |
| Alignment redundancy | 1.151 | 1.049 | 1.048 |
| Multi-mapping rate (%) | 13.1 | 4.7 | 4.8 |

**Supplementary Table 2.** Haplotype-phased HLA genes.

| Haplotype-phased HLA genes | Assembly-based HLA alleles | Alignment-based HLA alleles |
| --- | --- | --- |
| haplotype1.HLA-A | HLA-A*02:06:01:02 | HLA-A*02:06:01:02 |
| haplotype1.HLA-B | HLA-B*51:01:01:01 | HLA-B*51:01:01:01 |
| haplotype1.HLA-C | HLA-C*14:02:01:01 | HLA-C*14:02:01:01 |
| haplotype1.HLA-E | HLA-E*01:01:01:03 | HLA-E*01:01:01:03 |
| haplotype1.HLA-F | HLA-F*01:01:01:13 | HLA-F*01:01:01:13 |
| haplotype1.HLA-G | HLA-G*01:01:01:01 | HLA-G*01:01:01:01 |
| haplotype1.HLA-DRA | HLA-DRA*01:01:01:12 | HLA-DRA*01:01:01:12 |
| haplotype1.HLA-DRB1 | HLA-DRB1*09:59 | HLA-DRB1*07:01:01:15 |
| haplotype1.HLA-DRB5 | HLA-DRB5*01:01:01:02 | HLA-DRB5*01:01:01:02 |
| haplotype1.HLA-DQA1 | HLA-DQA1*03:02:01:01 | HLA-DQA1*03:02:01:01 |
| haplotype1.HLA-DQB1 | HLA-DQB1*03:03:02:03 | HLA-DQB1*03:01:01:01 |
| haplotype1.HLA-DQA2 | HLA-DQA2*01:01:02:01 | HLA-DQA2*01:01:01:03;HLA-DQA2*01:01:01:04;HLA-DQA2*01:01:01:06;HLA-DQA2*01:08 |
| haplotype1.HLA-DQB2 | HLA-DQB2*01:01:01:15 | HLA-DQB2*01:01:01:01 |
| haplotype1.HLA-DOB | HLA-DOB*01:01:01:24 | HLA-DOB*01:01:01:24 |
| haplotype1.HLA-DMB | HLA-DMB*01:07:01:04 | HLA-DMB*01:01:01:04 |
| haplotype1.HLA-DMA | HLA-DMA*01:03:01:02 | HLA-DMA*01:01:01:01 |
| haplotype1.HLA-DOA | HLA-DOA*01:01:04:01 | HLA-DOA*01:01:04:01 |
| haplotype1.HLA-DPA1 | HLA-DPA1*02:02:02:01 | HLA-DPA1*02:02:02:01 |
| haplotype1.HLA-DPB1 | HLA-DPB1*05:01:01:01 | HLA-DPB1*13:01:01:07 |
| haplotype2.HLA-A | HLA-A*02:06:01:01 | HLA-A*02:06:01:01 |
| haplotype2.HLA-B | HLA-B*40:01:02:04 | HLA-B*40:01:02:04 |
| haplotype2.HLA-C | HLA-C*07:02:01:15 | HLA-C*07:02:01:15 |
| haplotype2.HLA-E | HLA-E*01:03:02:01 | HLA-E*01:03:02:01;HLA-E*01:03:02:27;HLA-E*01:03:02:32;HLA-E*01:03:02:02;HLA-E*01:03:02:34;HLA-E*01:03:02:41;HLA-E*01:03:04;HLA-E*01:01:02;HLA-E*01:03:01:01;HLA-E*01:03:02:06;HLA-E*01:03:02:35;HLA-E*01:03:02:36;HLA-E*01:03:02:42 |
| haplotype2.HLA-F | HLA-F*01:01:01:13 | HLA-F*01:01:01:13 |
| haplotype2.HLA-G | HLA-G*01:01:01:01 | HLA-G*01:01:01:01 |
| haplotype2.HLA-DRA | HLA-DRA*01:01:01:12 | HLA-DRA*01:01:01:12 |
| haplotype2.HLA-DRB1 | HLA-DRB1*12:01:01:06 | HLA-DRB1*04:01:01:11 |
| haplotype2.HLA-DRB5 | HLA-DRB5*01:01:01:05 | HLA-DRB5*01:01:01:05 |
| haplotype2.HLA-DQA1 | HLA-DQA1*05:05:01:03 | HLA-DQA1*05:05:01:03 |
| haplotype2.HLA-DQB1 | HLA-DQB1*03:01:01:01 | HLA-DQB1*03:01:01:01 |
| haplotype2.HLA-DQA2 | HLA-DQA2*01:01:02:01 | HLA-DQA2*01:01:02:01 |
| haplotype2.HLA-DQB2 | HLA-DQB2*01:01:01:01 | HLA-DQB2*01:01:01:15;HLA-DQB2*01:01:01:03;HLA-DQB2*01:01:01:04;HLA-DQB2*01:01:01:06;HLA-DQB2*01:05;HLA-DQB2*01:01:01:10;HLA-DQB2*01:01:01:12;HLA-DQB2*01:03;HLA-DQB2*01:01:01:02;HLA-DQB2*01:01:01:08;HLA-DQB2*01:01:01:09;HLA-DQB2*01:01:01:16;HLA-DQB2*01:01:01:17;HLA-DQB2*01:01:03;HLA-DQB2*01:01:01:05;HLA-DQB2*01:01:01:07;HLA-DQB2*01:01:01:11;HLA-DQB2*01:01:01:13;HLA-DQB2*01:01:01:14;HLA-DQB2*01:01:01:18;HLA-DQB2*01:02:01:01;HLA-DQB2*01:02:01:02;HLA-DQB2*01:02:01:03;HLA-DQB2*01:02:01:04;HLA-DQB2*01:02:02:01;HLA-DQB2*01:02:02:02;HLA-DQB2*01:02:02:03;HLA-DQB2*01:02:02:04;HLA-DQB2*01:02:02:05;HLA-DQB2*01:02:04 |
| haplotype2.HLA-DOB | HLA-DOB*01:01:01:24 | HLA-DOB*01:01:01:24 |
| haplotype2.HLA-DMB | HLA-DMB*01:01:01:01 | HLA-DMB*01:01:01:04 |
| haplotype2.HLA-DMA | HLA-DMA*01:01:01:01 | HLA-DMA*01:01:01:02;HLA-DMA*01:01:01:04 |
| haplotype2.HLA-DOA | HLA-DOA*01:01:04:01 | HLA-DOA*01:01:04:01 |
| haplotype2.HLA-DPA1 | HLA-DPA1*02:02:02:01 | HLA-DPA1*02:02:02:01 |
| haplotype2.HLA-DPB1 | HLA-DPB1*05:01:01:01 | HLA-DPB1*13:01:01:07 |

**Supplementary Table 3. Summary of assembly quality metrics for COLO829BL.**

| Statistics | COLO829BL personalized assembly |
| --- | --- |
| Assembled bases (Gb) | 3.09 |
| Unplaced bases (Mb) | 24.72 |
| Gaps | 56 |
| Number of contigs | 173 |
| Contig N50 (Mb) | 91.78 |
| Number of scaffolds | 105 |
| Scaffold N50 (Mb) | 150.66 |
| QV | 66.53 |
| Completeness (%) | 97.73 |
| BUSCO (%) | 96.1 |
| Genome fraction (%) (compare to GRCh38) | 97.47 |
| Correctly assembled (%) | 98.31 |
| Erroneous (%) | 1.14 |

**Supplementary Table 4.** **Small-variant calling results across three reference genomes.**

| Genome | Caller/Consensus | VCF-liftover | | | BAM-liftover | | |
| --- | --- | --- | --- | --- | --- | --- | --- |
|  |  | SNPs | INDELs | Total | SNPs | INDELs | Total |
| GRCh38 | deepSomatic | 41570 | 834 | 42404 | 41570 | 834 | 42404 |
|  | ClairS | 38980 | 871 | 39851 | 38980 | 871 | 39851 |
|  | Consensus | 37950 | 455 | 38405 | 37950 | 455 | 38405 |
| pGenome | deepSomatic | 43788 | 999 | 44787 | 41159 | 831 | 41990 |
|  | ClairS | 44979 | 1272 | 46251 | 38912 | 891 | 39803 |
|  | Consensus | 40233 | 532 | 40765 | 37845 | 445 | 38290 |
| T2T-CHM13 | deepSomatic | 42414 | 856 | 43270 | 41174 | 825 | 41999 |
|  | ClairS | 41269 | 961 | 42230 | 39003 | 892 | 39895 |
|  | Consensus | 39027 | 473 | 39500 | 37852 | 440 | 38292 |

**Supplementary Table 5.** **Somatic SNV benchmarking across the three genomes using VCF-liftover and BAM-liftover.**

| Genome | Truth Count | Call Count | TP | FP | FN | Precision | Recall | F1 Score | Method |
| --- | --- | --- | --- | --- | --- | --- | --- | --- | --- |
| GRCh38 | 36649 | 37950 | 35605 | 2345 | 1044 | 0.938 | 0.972 | 0.955 | VCF-liftover |
| T2T-CHM13 | 36488 | 39027 | 35433 | 3594 | 1055 | 0.908 | 0.971 | 0.938 |  |
| pGenome | 36337 | 40233 | 35454 | 4779 | 883 | 0.881 | 0.976 | 0.926 |  |
| GRCh38 | 36649 | 37950 | 35605 | 2345 | 1044 | 0.938 | 0.972 | 0.955 | BAM-liftover |
| T2T-CHM13 | 36649 | 37845 | 35479 | 2366 | 1170 | 0.937 | 0.968 | 0.953 |  |
| pGenome | 36649 | 37852 | 35465 | 2387 | 1184 | 0.937 | 0.968 | 0.952 |  |

**Supplementary Table 6.** **Structural variant calling results across three reference genomes.**

| Genome | Caller/Consensus | VCF-liftover | BAM-liftover |
| --- | --- | --- | --- |
| GRCh38 | SAVANA | 152 | NA |
|  | Severus | 110 | 110 |
|  | nanomonsv | 106 | 106 |
|  | Sniffles2 | 210 | 210 |
|  | SVision-pro | 1707 | 1707 |
|  | Consensus | 74 | 73 |
| T2T-CHM13 | SAVANA | 165 | NA |
|  | Severus | 110 | 106 |
|  | nanomonsv | 111 | 84 |
|  | Sniffles2 | 443 | 228 |
|  | SVision-pro | 497 | 375 |
|  | Consensus | 82 | 63 |
| pGenome | SAVANA | 205 | NA |
|  | Severus | 95 | 103 |
|  | nanomonsv | 124 | 81 |
|  | Sniffles2 | 262 | 232 |
|  | SVision-pro | 494 | 369 |
|  | Consensus | 90 | 63 |

**Supplementary Table 7.** **Somatic SV benchmarking across the three genomes using VCF-liftover and BAM-liftover.**

| Genome | Truth Count | Call Count | TP | FP | FN | Precision | Recall | F1 Score | Method |
| --- | --- | --- | --- | --- | --- | --- | --- | --- | --- |
| GRCh38 | 68 | 57 | 49 | 8 | 19 | 0.860 | 0.721 | 0.784 | VCF-liftover |
| T2T-CHM13 | 49 | 60 | 42 | 18 | 7 | 0.700 | 0.857 | 0.771 |  |
| pGenome | 67 | 71 | 48 | 23 | 19 | 0.676 | 0.716 | 0.696 |  |
| GRCh38 | 68 | 73 | 50 | 23 | 18 | 0.685 | 0.735 | 0.709 | BAM-liftover |
| T2T-CHM13 | 68 | 63 | 51 | 12 | 17 | 0.810 | 0.750 | 0.779 |  |
| pGenome | 68 | 63 | 51 | 12 | 17 | 0.810 | 0.750 | 0.779 |  |

**Supplementary Table 8.** Primer sequences used for PCR validation.

| Mutation type | Gene/ID | Locus | Forward | Reverse |
| --- | --- | --- | --- | --- |
| SNV | SNX29 | chr16:12558207-12559008 | TTGCGTTGCTCTGTGTTGGC | ACCCTGGTGTGGAGATGCAC |
| SNV | TFPT | chr19:58701826-58702627 | TCTCAGATCTGCCACTGCTG | TCCCAGGTTCAAGCAGTCCT |
| SNV | CDH4 | chr20:62265593-62266394 | TCCGTAGACATGCACCATGA | ATGCCCCAGCTGTGGTTTCT |
| SNV | RAC2 | chr22:31033454-31034255 | TGAGGCTCAGAGAGGATGTC | CTGCCTCAGCTTGATGAGCC |
| SNV | MDS2 | chr1:25833909-25834710 | TAGGTTCAGATGATGTGGGT | AGGGCTGAGAAGAGGTCACT |
| SNV | PRRX1 | chr1:183542626-183543427 | TGGATATAATAGATCCCTAA | AGCAGAATCTTGGAAGAAAG |
| SNV | SMYD3 | chr1:259535654-259536455 | TCCTCTCTTCCAGGCTGAAC | ACTCCTACTTCTAGGAGTTT |
| SNV | ADARB2 | chr10:1214063-1214864 | GGAATTCTGGTTTGTGCATAATGGA | ATGGAAGAAGGGTCCTGCACGAGTG |
| SNV | COL2A1 | chr12:50044006-50044807 | CCTGTGTGGGGAGAGGAGAGCCCCT | TGAGTGAGGCCTCTGACACCCCACC |
| SNV | PAX5 | chr9:37003733-37004534 | CAGCTACAGAACACACAACGGCCTC | GGTGTATACCGAAGAGTCTTGGGTT |
| SNV | RASGEF1A | chr10:45890110-45890911 | CTGAAATGTCAGTGTCTGCACACAG | GTCCTTCAGGTGCATGGCATTCACC |
| SNV | AKT3 | chr1:257208495-257209296 | TGCTAAATTAGAGAAATTCT | AGTAGATGTTCAGCCCCTTG |
| SV | Minda_93 | chr13:17413939-17414137 | AGTACACTCCAACCCCCTCC | GAATGGAGGGGAGGGTTTGT |
| SV | Minda_176 | chr17:80360942-80361046 | TGGCCCGTAAAGCTCCAAAT | GCAGAAAGGGGAGGTGTGAA |
| SV | Minda_178 | chr17:82458560-82458741 | AGCAGGAATGGGAGTTGTCC | CCACTTCCTCTCCCTCCACT |
| SV | Minda_248 | chr22:1010576-1010644 | AATTTCCCTGTCGGCAGAGG | TCGCTGAGGTGATGCAGAAC |

**Supplementary Table 9.** Primer sequences used for dual-luciferase reporter assay.

| Gene | Forward | Reverse |
| --- | --- | --- |
| MET-enhancer | TACGGTACCCATGATGAATTGAACCCATTTATTA | GTAGAGCTCGTTCTGTGCAAAATACTTTACAAA |
| MET-promoter | TACCTCGAGACCTTCACACACCCAGATAC | GTATTCGAACTATAAAAATTGGCACCTACAAGAG |
| HSD17B2-silencer | TACGAGCTCTGGAGTGCAGTGGCGTGATC | GTACTCGAGTCAAGCAATCCACCTGACCTC |
| HSD17B2-promoter | TACCTCGAGCGAAGAGCCATAGCTAGTCC | GTAGATATCCAGTGGAGTCTTGCAGCCTG |

**Supplementary Table 10. Practical guidance for selecting benchmarking and validation strategies in personalized-reference somatic variant analysis**

| Strategy | Recommended role | Best suited for | Main advantage | Key limitation | Practical recommendation |
| --- | --- | --- | --- | --- | --- |
| BAM-liftover | Recommended for GRCh38-anchored benchmarking and GRCh38-based annotation | Standardized comparison across references when the available truth set or downstream annotation framework is GRCh38-based | Allows reads to benefit from alignment to T2T-CHM13 or pGenome while somatic variant calling, benchmarking, and annotation are performed in GRCh38 coordinates | May not fully evaluate variants located in personalized, non-GRCh38, or poorly liftable regions; depends on successful alignment liftover | Use for fair performance benchmarking across references and for GRCh38-centered downstream interpretation; not a universal replacement for personalized-reference discovery |
| VCF-liftover | Recommended for personalized-reference discovery and reference-specific analysis | Analyses aiming to call variants directly on each reference genome, including patient-specific pGenome-based HCC analyses | Preserves native alignment, variant-calling context, and pGenome coordinate information, which can reveal variants in individualized, repetitive, or complex regions | Apparent false positives may reflect incompleteness or reference bias of GRCh38-based truth sets rather than technical errors; variant representation can complicate benchmarking | Use for discovery-oriented analyses; interpret apparent false positives cautiously and support key findings with read-level, orthogonal, or assembly-based validation |
| Direct assembly-based comparison | Orthogonal validation and structural interpretation | Resolving discordant calls or candidate variants in repetitive, centromeric, complex, or pGenome-specific regions | Provides assembly-level evidence that is less dependent on GRCh38-based truth-set completeness and is useful for complex SV interpretation | Requires high-quality tumor and normal assemblies; may be less sensitive for low-VAF or subclonal variants and is not ideal as a standalone high-throughput benchmark metric | Use as a validation layer for ambiguous, novel, repetitive-region, or pGenome-specific variants, especially when VCF-liftover and BAM-liftover results differ |

*Note. These strategies are complementary: BAM-liftover is most appropriate for GRCh38-anchored benchmarking and annotation; VCF-liftover is appropriate for personalized-reference discovery; direct assembly-based comparison provides orthogonal validation for ambiguous or complex variants.*

**Supplementary Table 11. Raw dual-luciferase reporter assay measurements**

| Gene | Cell line | ConstructGroup | Replicate | Firefly RLU | Renilla RLU | Firefly/Renilla | Fold change | FDR | % change |
| --- | --- | --- | --- | --- | --- | --- | --- | --- | --- |
| MET | 293T | pGL4.10-Basic | 1;2;3 | 72;87;92 | 26132;27199;28352 | 0.003;0.003;0.003 | 1.275058 | 0.025925935 | 27.50579 |
|  |  | Promoter-only | 1;2;3 | 2847;3218;3002 | 27018;29521;27763 | 0.105;0.109;0.108 |  |  |  |
|  |  | Wild-type enhancer | 1;2;3 | 4721;4957;4696 | 29883;30028;30489 | 0.158;0.165;0.154 |  |  |  |
|  |  | Mutant enhancer | 1;2;3 | 5848;5672;5796 | 26722;28877;30024 | 0.219;0.196;0.193 |  |  |  |
|  | HepG2 | pGL4.10-Basic | 1;2;3 | 28;19;24 | 17382;16928;16390 | 0.002;0.001;0.001 | 1.28126 | 0.010642959 | 28.12596 |
|  |  | Promoter-only | 1;2;3 | 1271;1347;1439 | 12785;13342;13002 | 0.099;0.101;0.111 |  |  |  |
|  |  | Wild-type enhancer | 1;2;3 | 2017;2119;2140 | 14341;14110;14829 | 0.141;0.150;0.144 |  |  |  |
|  |  | Mutant enhancer | 1;2;3 | 2611;2490;2418 | 14112;12713;13689 | 0.185;0.196;0.177 |  |  |  |
|  | Huh7 | pGL4.10-Basic | 1;2;3 | 27;37;29 | 18731;17842;16390 | 0.001;0.002;0.002 | 1.165932 | 0.00165563 | 16.59319 |
|  |  | Promoter-only | 1;2;3 | 1124;1275;1216 | 16910;17376;16533 | 0.066;0.073;0.074 |  |  |  |
|  |  | Wild-type enhancer | 1;2;3 | 1143;1124;1103 | 13791;14071;13653 | 0.083;0.080;0.081 |  |  |  |
|  |  | Mutant enhancer | 1;2;3 | 1439;1398;1477 | 15067;14632;15897 | 0.096;0.096;0.093 |  |  |  |
| HSD17B2 | 293T | pGL4.10-Basic | 1;2;3 | 72;87;92 | 26132;27199;28352 | 0.003;0.003;0.003 | 0.735099 | 0.007071738 | -26.4901 |
|  |  | Promoter-only | 1;2;3 | 3462;3600;3455 | 34725;34858;34007 | 0.100;0.103;0.102 |  |  |  |
|  |  | Wild-type silencer | 1;2;3 | 4430;4318;4327 | 99311;96185;106941 | 0.045;0.045;0.040 |  |  |  |
|  |  | Mutant silencer | 1;2;3 | 2275;2370;1293 | 75097;76852;37585 | 0.030;0.031;0.034 |  |  |  |
|  | HepG2 | pGL4.10-Basic | 1;2;3 | 23;21;31 | 18671;16923;17392 | 0.001;0.001;0.002 | 0.705676 | 0.002261885 | -29.4324 |
|  |  | Promoter-only | 1;2;3 | 2114;2106;2004 | 12014;12476;12314 | 0.176;0.169;0.163 |  |  |  |
|  |  | Wild-type silencer | 1;2;3 | 1602;1321;1254 | 12385;9836;10132 | 0.129;0.134;0.124 |  |  |  |
|  |  | Mutant silencer | 1;2;3 | 1064;1108;1097 | 11935;11454;12536 | 0.089;0.097;0.088 |  |  |  |
|  | Huh7 | pGL4.10-Basic | 1;2;3 | 20;23;29 | 18319;16794;17841 | 0.001;0.001;0.002 | 0.764415 | 0.010642959 | -23.5585 |
|  |  | Promoter-only | 1;2;3 | 1701;1843;1738 | 11843;12318;11932 | 0.144;0.150;0.146 |  |  |  |
|  |  | Wild-type silencer | 1;2;3 | 1329;1298;1304 | 12901;13014;12917 | 0.103;0.100;0.101 |  |  |  |
|  |  | Mutant silencer | 1;2;3 | 910;991;902 | 11701;12093;12452 | 0.078;0.082;0.072 |  |  |  |

Note: Fold change, FDR, and % change indicate the mutant versus wild-type fragment comparison within each gene and cell line. These values are shown once for each gene–cell line comparison and apply to the corresponding wild-type and mutant fragment pair.

**Supplementary Table 12. Summary of sample quantity and quality metrics for genomic, transcriptomic, and epigenomic experiments.**

| Experiment | Sample / library ID | Input material | Sample input / available amount | Qubit concentration (ng/µL) | Purity / integrity metrics |
| --- | --- | --- | --- | --- | --- |
| HiFi | Normal | DNA | 81.868 µg available DNA | 422 | A260/280: 1.89; A260/230: 2.41 |
| HiFi | Tumor | DNA | 78.960 µg available DNA | 560 | A260/280: 1.86; A260/230: 2.35 |
| ONT | Normal | DNA | 21.600 µg available DNA | 160 | A260/280: 1.87; A260/230: 2.44 |
| ONT | Tumor | DNA | 24.120 µg available DNA | 268 | A260/280: 1.89; A260/230: 2.40 |
| HiC | Normal | Tissue / nuclei | 50 mg tissue; 1×10⁶ nuclei;  estimated DNA yield: 9.88 µg | 49.4 | A260/280: 1.84; A260/230: 2.36 |
| HiC | Tumor | Tissue / nuclei | 50 mg tissue; 1×10⁶ nuclei;  estimated DNA yield: 3.38 µg | 16.9 | A260/280: 1.83; A260/230: 2.41 |
| RNA-seq | normal_rep1 | Total RNA | 9.010 µg available total RNA | 170 | A260/280: 2.03; A260/230: 2.12; RIN: 7.6 |
| RNA-seq | normal_rep2 | Total RNA | 8.374 µg available total RNA | 158 | A260/280: 2.04; A260/230: 2.15; RIN: 7.5 |
| RNA-seq | tumor_rep1 | Total RNA | 11.289 µg available total RNA | 213 | A260/280: 2.02; A260/230: 2.05; RIN: 7.1 |
| RNA-seq | tumor_rep2 | Total RNA | 11.978 µg available total RNA | 226 | A260/280: 2.01; A260/230: 2.08; RIN: 7.3 |
| CUT&Tag | Normal/Tumor, H3K4me1, H3K4me3, H3K27me3, H3K27ac | Tissue / nuclei | 20 mg tissue; 5×10⁴ nuclei per assay | — | — |

**Supplementary Figures**

**
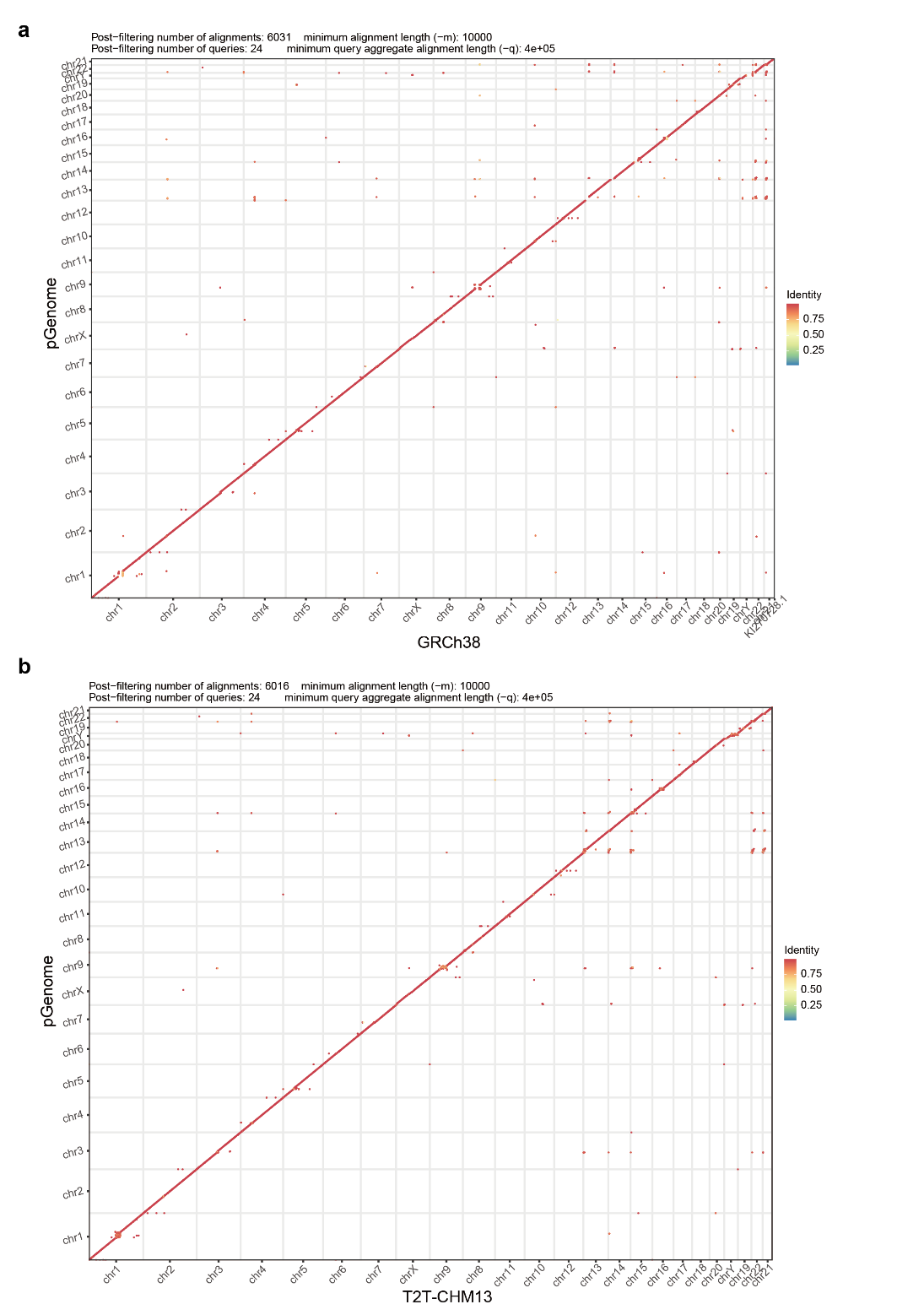
**

**Supplementary Figure 1. Genome collinearity analysis.** Dot plots illustrating whole-genome alignment between pGenome and (**a**) GRCh38 or (**b**) T2T-CHM13 reference genomes. Red dots represent syntenic regions with sequence similarity >90%.


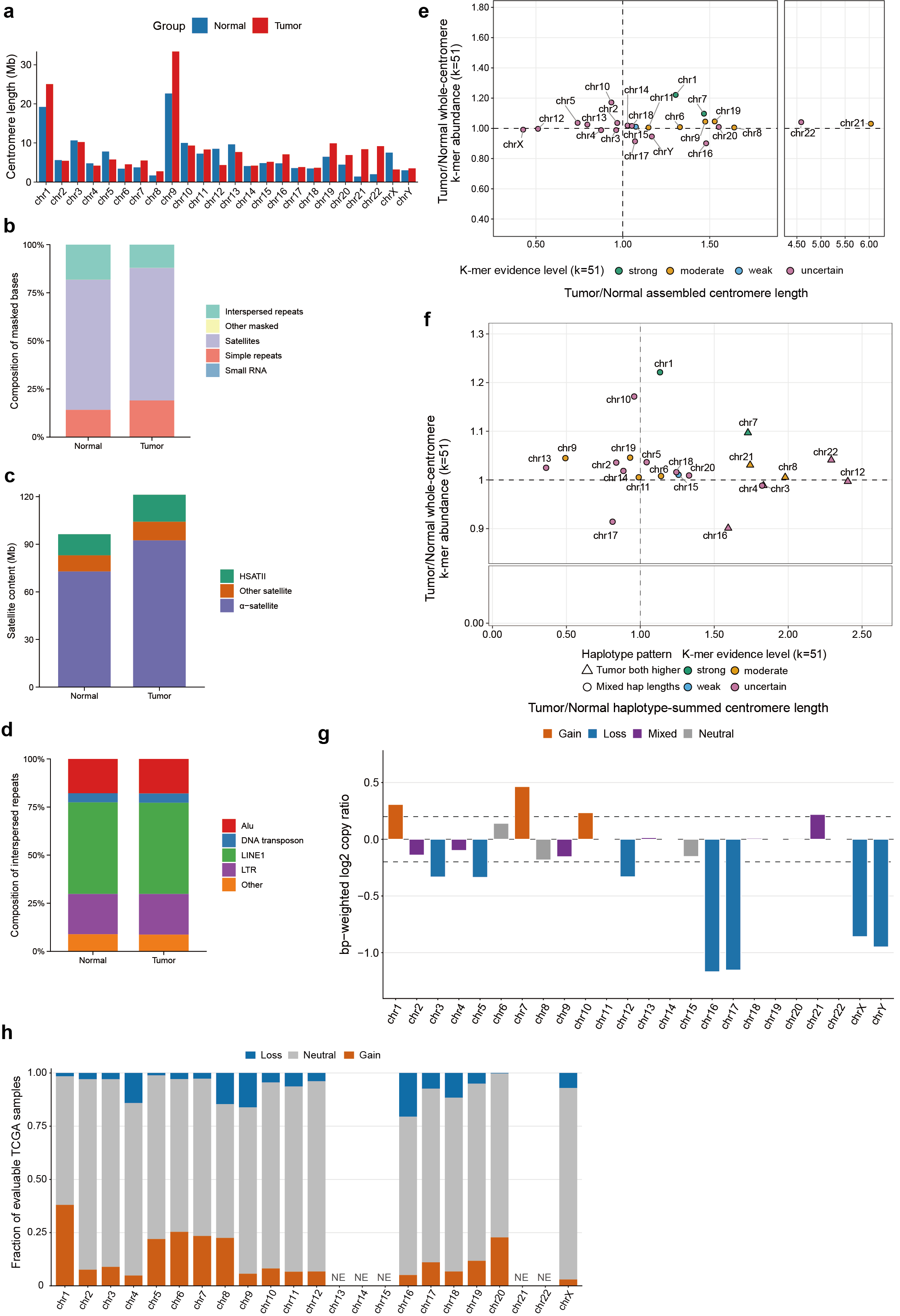


**Supplementary Figure 2.** Patient-specific centromeric remodeling and cohort-level centromeric CNV context. **a,** Chromosome-level assembled centromere lengths in matched normal and tumor personalized genomes. **b,** Repeat class composition within de novo assembled centromeric regions. **c,** Satellite subclass content within assembled centromeric regions. α-satellite corresponds to ALR/Alpha annotations. **d,** Relative contribution of interspersed repeat classes within assembled centromeric regions. **e,** Comparison of tumor/normal assembled centromere length ratios and whole-centromere k-mer abundance ratios using k = 51. The x-axis shows tumor/normal assembled centromere length ratios, and the y-axis shows tumor/normal whole-centromere k-mer abundance ratios. Dashed lines indicate a ratio of 1. Colors indicate k-mer support levels. **f,** Haplotype-aware comparison of tumor/normal assembled centromere length ratios and whole-centromere k-mer abundance ratios using k = 51. The x-axis shows haplotype-summed assembled centromere length ratios, and the y-axis shows whole-centromere k-mer abundance ratios. Dashed lines indicate a ratio of 1. Point shapes indicate haplotype-level length patterns, and colors indicate k-mer support levels. chr1 showed a tumor-higher signal with concordant k-mer support in both comparisons. **g,** Bp-weighted centromeric CNV summary in the tumor genome relative to the matched normal genome. CNV segments overlapping each centromere were summarized using bp-weighted mean log2 copy ratio. Horizontal dashed lines indicate gain/loss thresholds. Chromosomes are colored as gain, loss, mixed, or neutral; mixed denotes centromeres containing both gain and loss segments. **h,** Fraction of evaluable TCGA-LIHC samples classified as gain, loss, or neutral for each centromeric region. NE denotes centromeres that could not be robustly evaluated. This cohort-level analysis provides centromeric CNV context but does not directly measure centromeric array length.


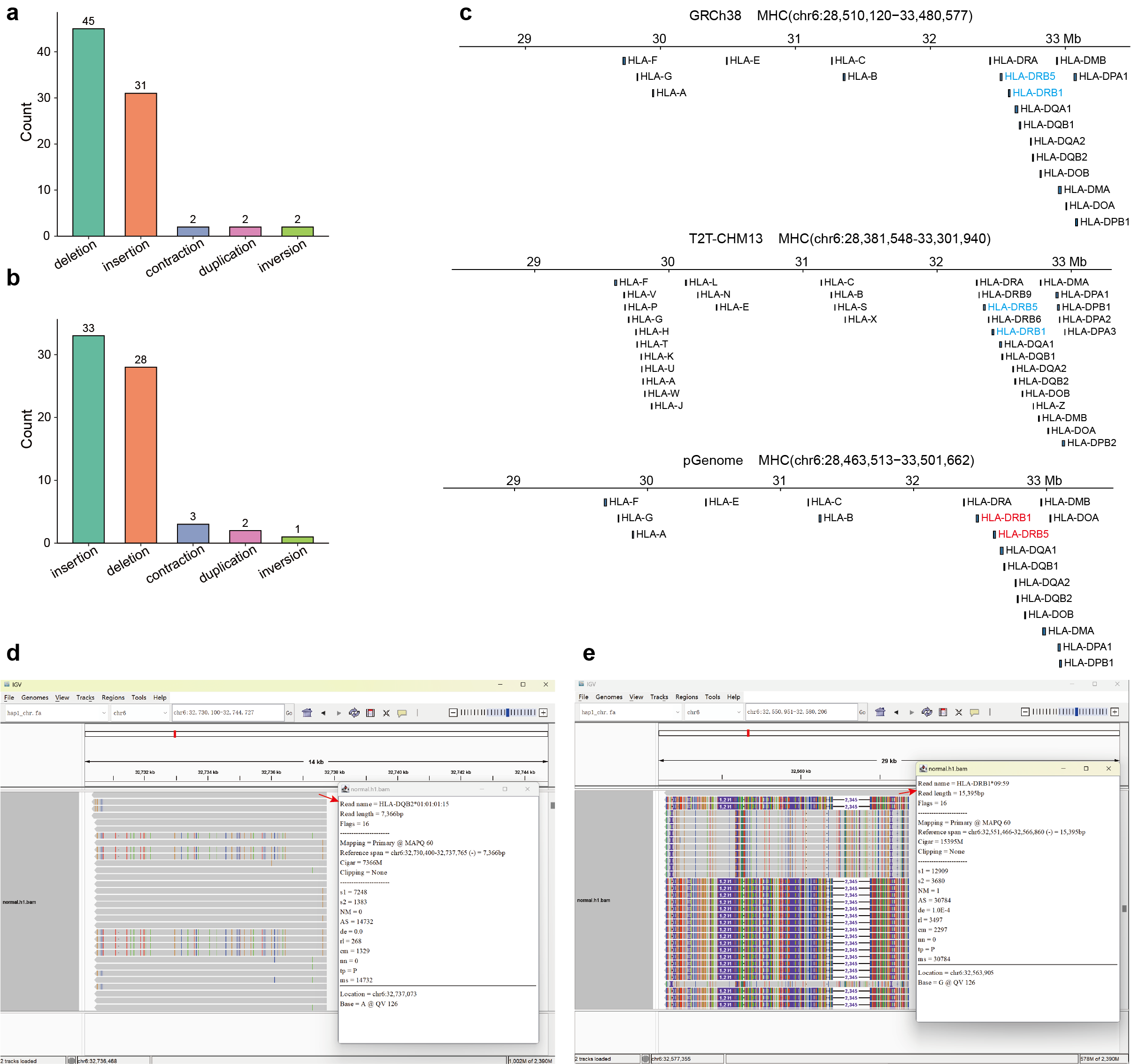


**Supplementary Figure 3. Comparative and haplotype-resolved analysis of the MHC region. a**, Structural variations in the MHC region of pGenome relative to GRCh38. **b**, Structural variations in the MHC region of pGenome relative to T2T-CHM13. “Contraction” denotes tandem contraction, defined as a reduction in tandem repeat copy number relative to the reference genome. **c**, Comparative annotation of HLA genes within MHC regions of GRCh38, T2T-CHM13 and pGenome. **d,** IGV visualization of IMGT/HLA allele sequences aligned to the normal haplotype 1 assembly at the HLA-E and DQB2 loci. **e,** IGV visualization of IMGT/HLA allele sequences aligned to the normal haplotype 1 assembly. HLA-DRB1*09:59 shows the highest concordance.


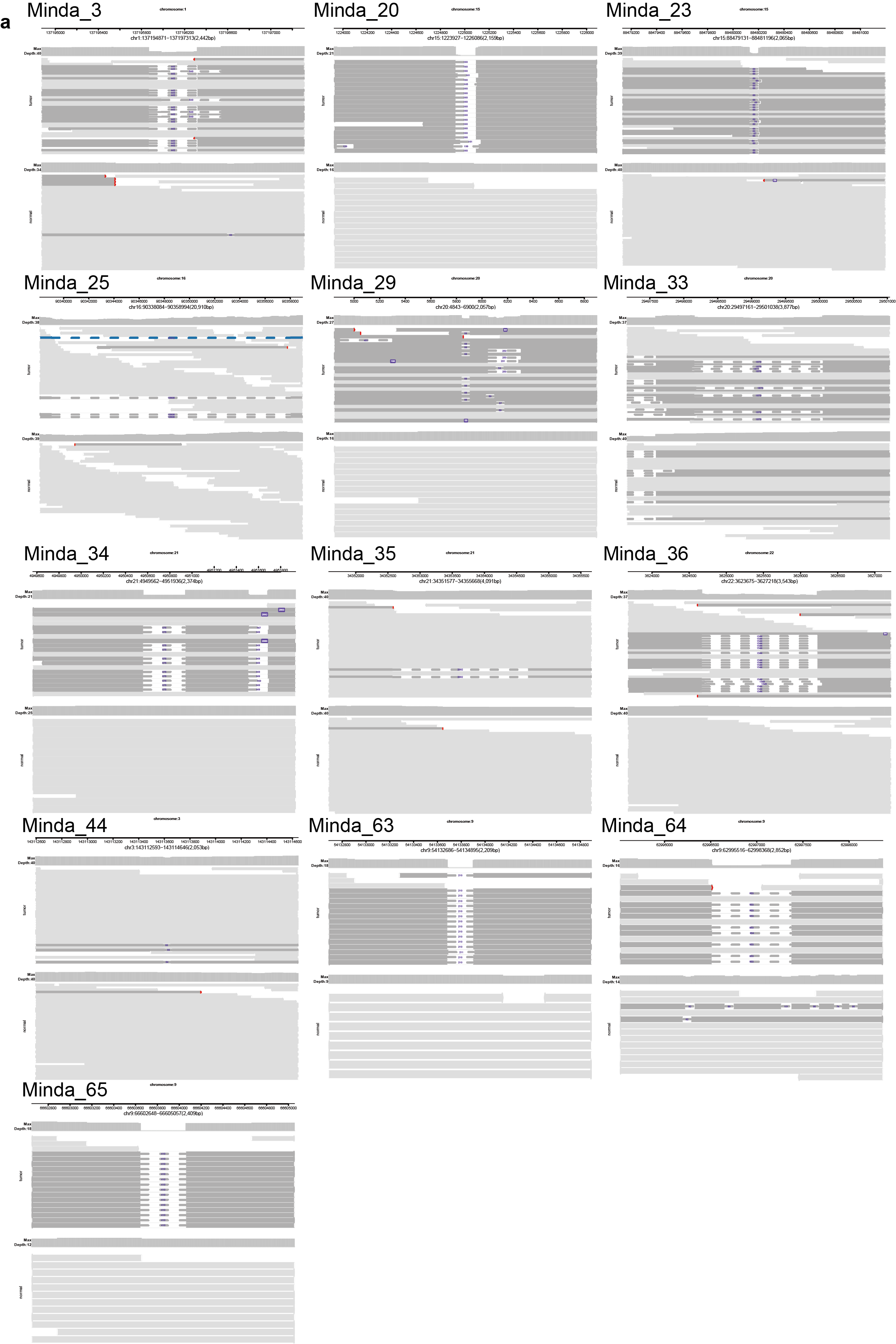


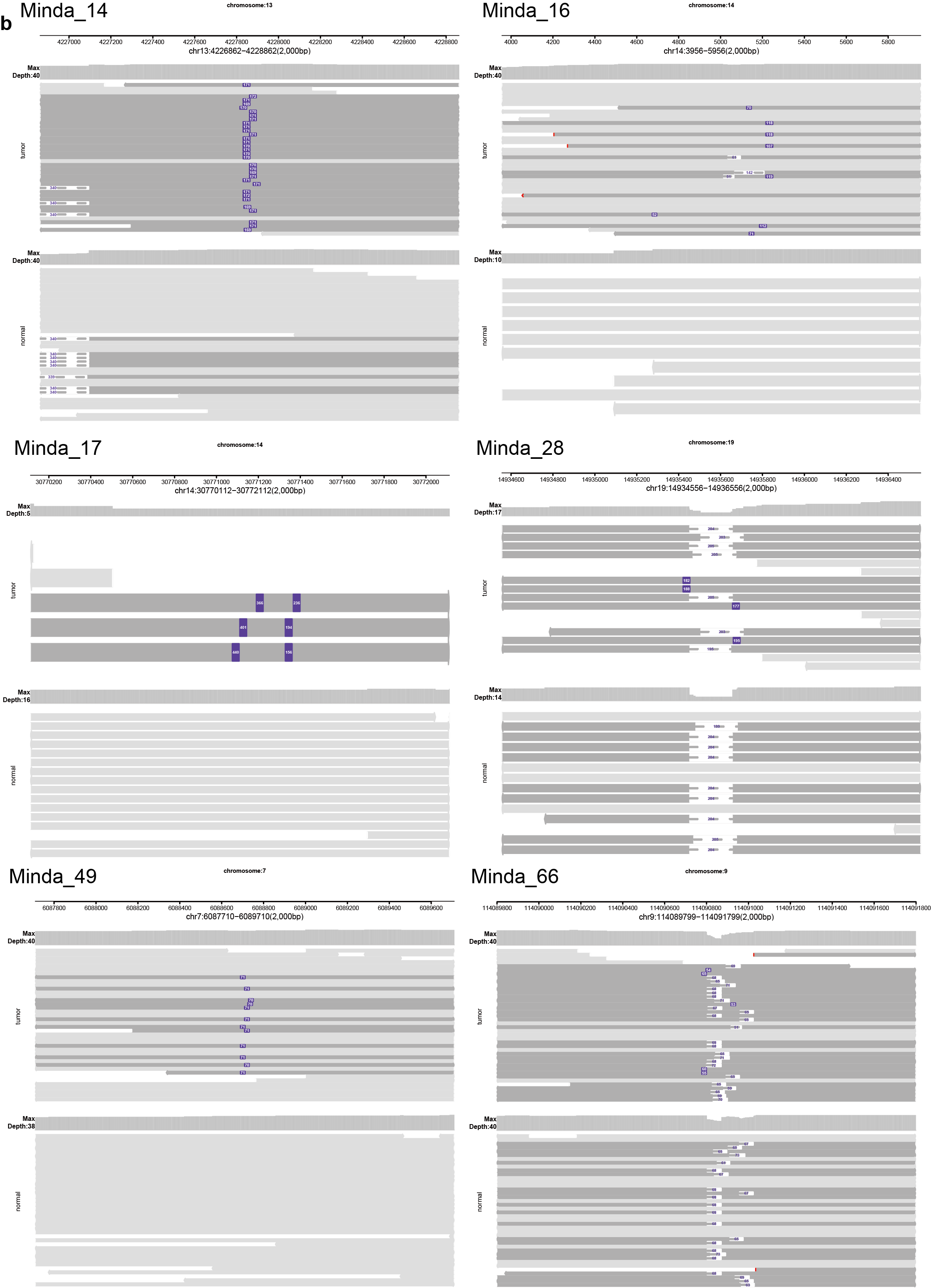


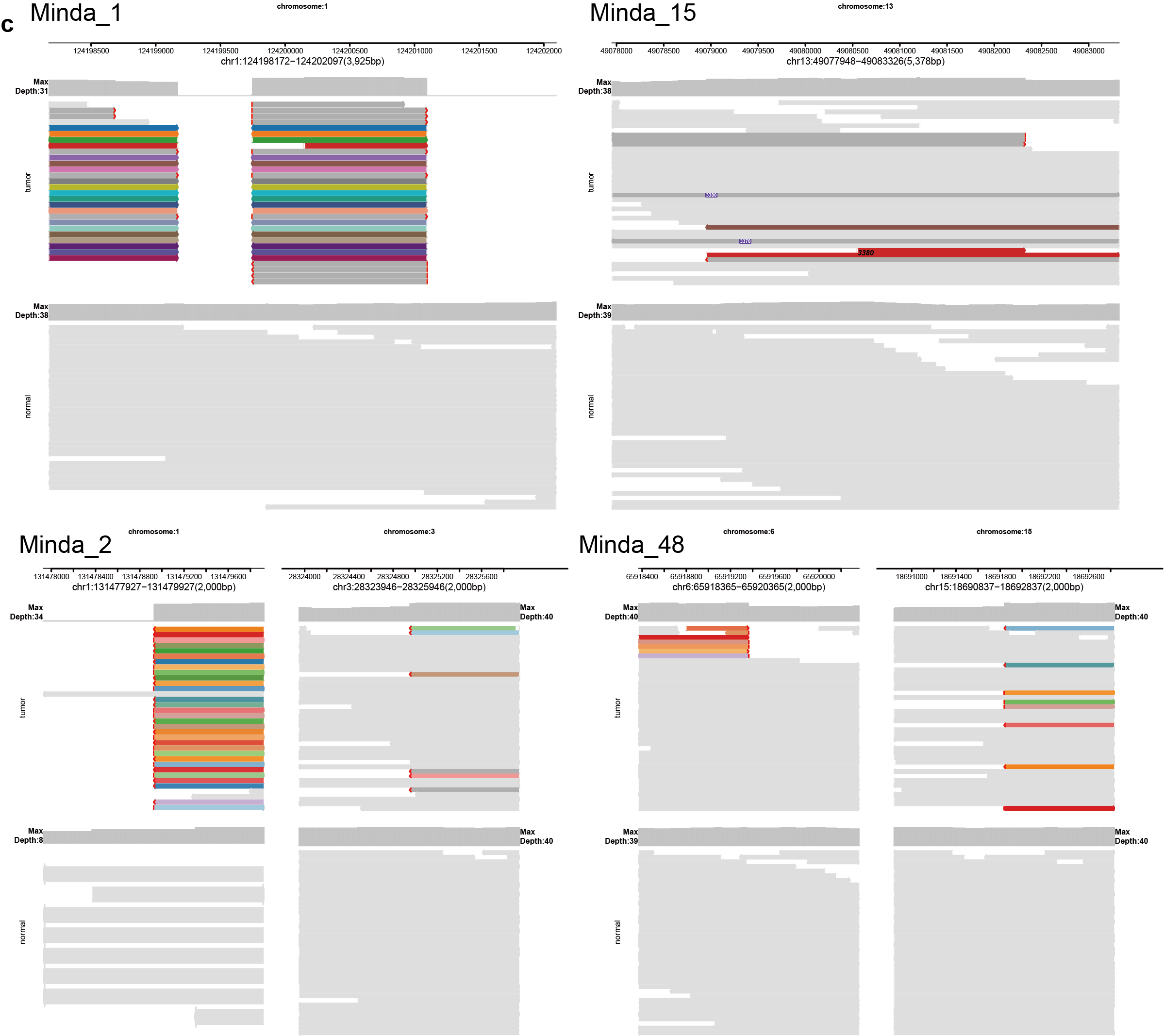


**Supplementary Figure 4. Genome-browser validation of 23 “false-positive” SVs in pGenome.** (a) Deletions (n = 13). (b) Insertions (n = 6). (c) Inversions, duplications, and translocations (n = 4). For each SV, the upper panel shows the tumor sample and the lower panel shows the matched normal sample.


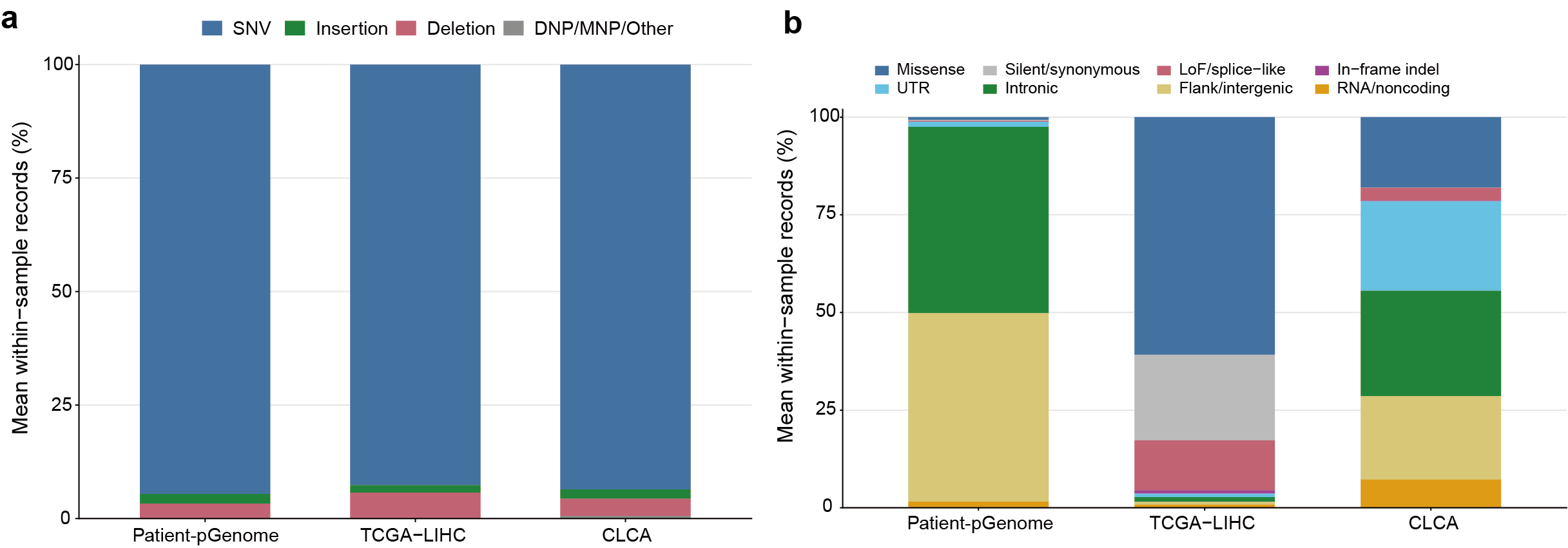


**Supplementary Figure 5. Cohort-level context for somatic small-variant composition.**

**a,** Variant-type composition of somatic variants in the patient pGenome-based calls, TCGA-LIHC, and CLCA. **b,** Functional annotation composition of somatic variants across the same datasets.


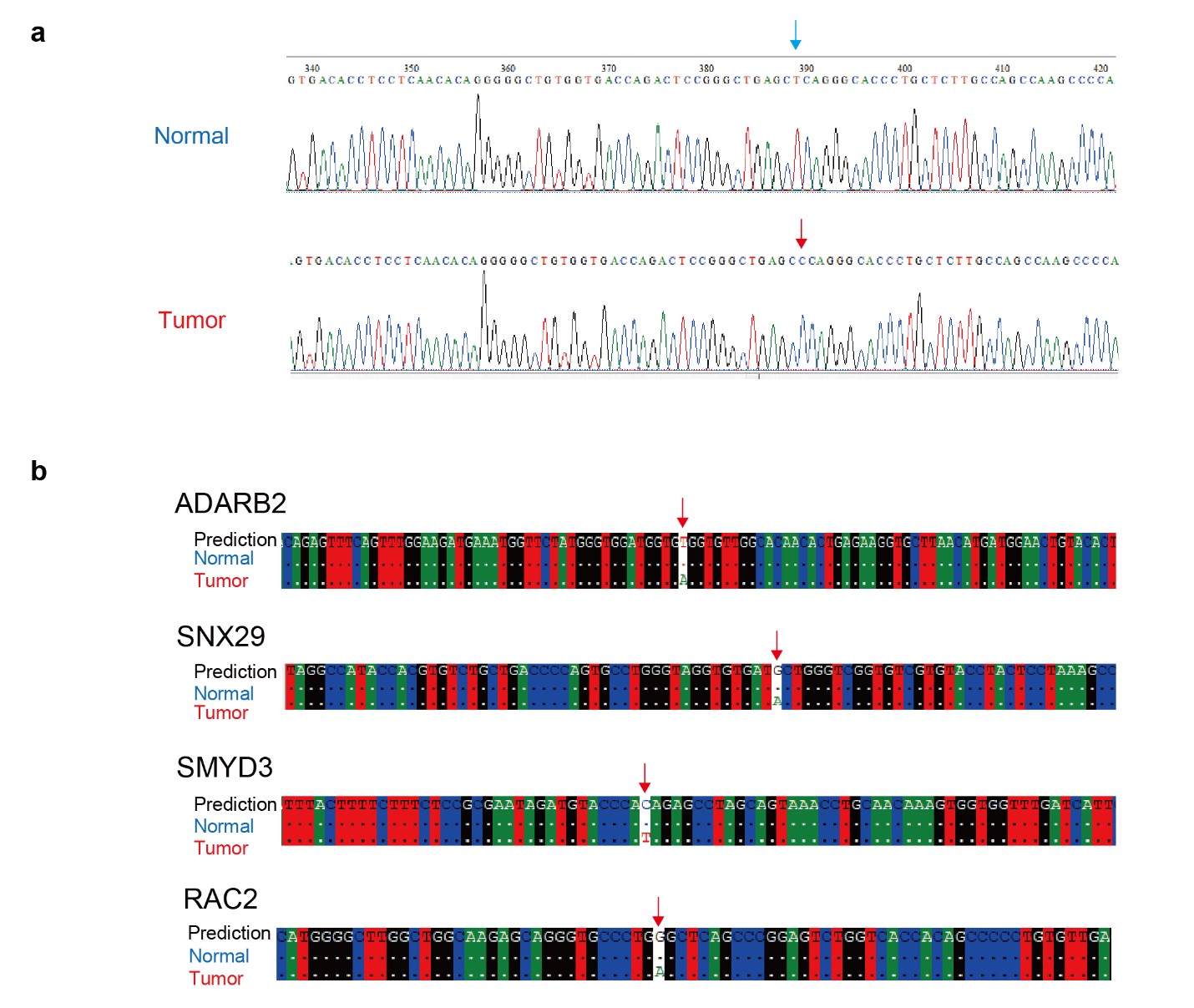


**Supplementary Figure 6. Representative Sanger validation of somatic SNVs.** **a,** Representative Sanger chromatogram showing validation of a somatic SNV; the arrow indicates the mutated base. **b**, Validated examples include *ADARB2* (chr10:1214063–1214864 T>A), *SNX29* (chr16:12558207–12559008 C>T), *SMYD3* (chr1:259535654–259536455 C>T), and *RAC2* (chr22:31033454–31034255 G>A).


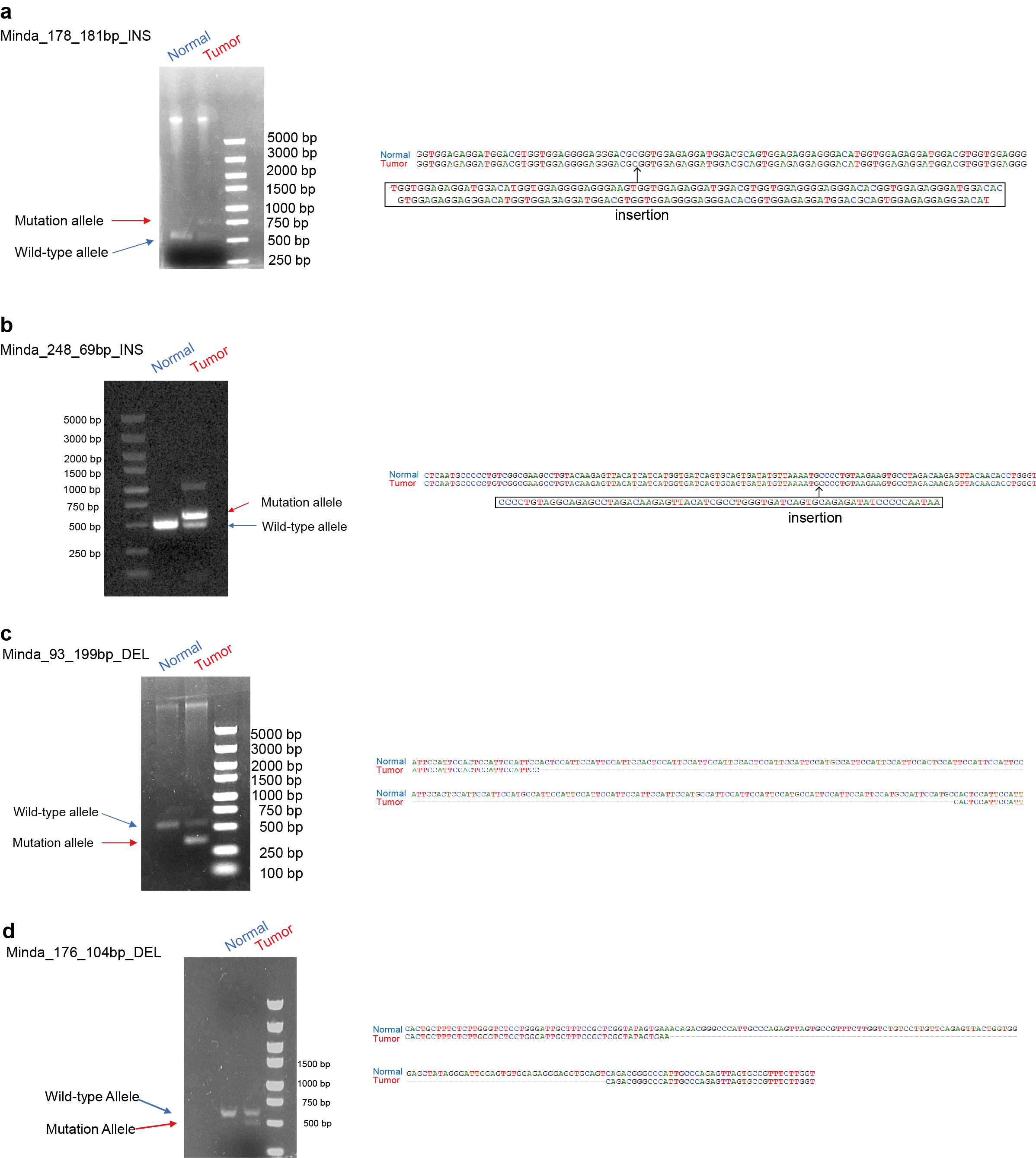


**Supplementary Figure 7. Experimental validation of representative pGenome-unique SVs.**PCR and Sanger sequencing validation of four representative pGenome-unique SVs selected from distinct reference-dependent categories. **a,** Minda_178, representing an SV type mismatch. **b,** Minda_248, representing an unmapped/fully mappable breakpoint pattern. **c,** Minda_93, representing an unmapped/unmapped breakpoint pattern. **d,** Minda_176, representing a complex breakpoint-mappability case involving multiple distant alignments in the reference genomes.


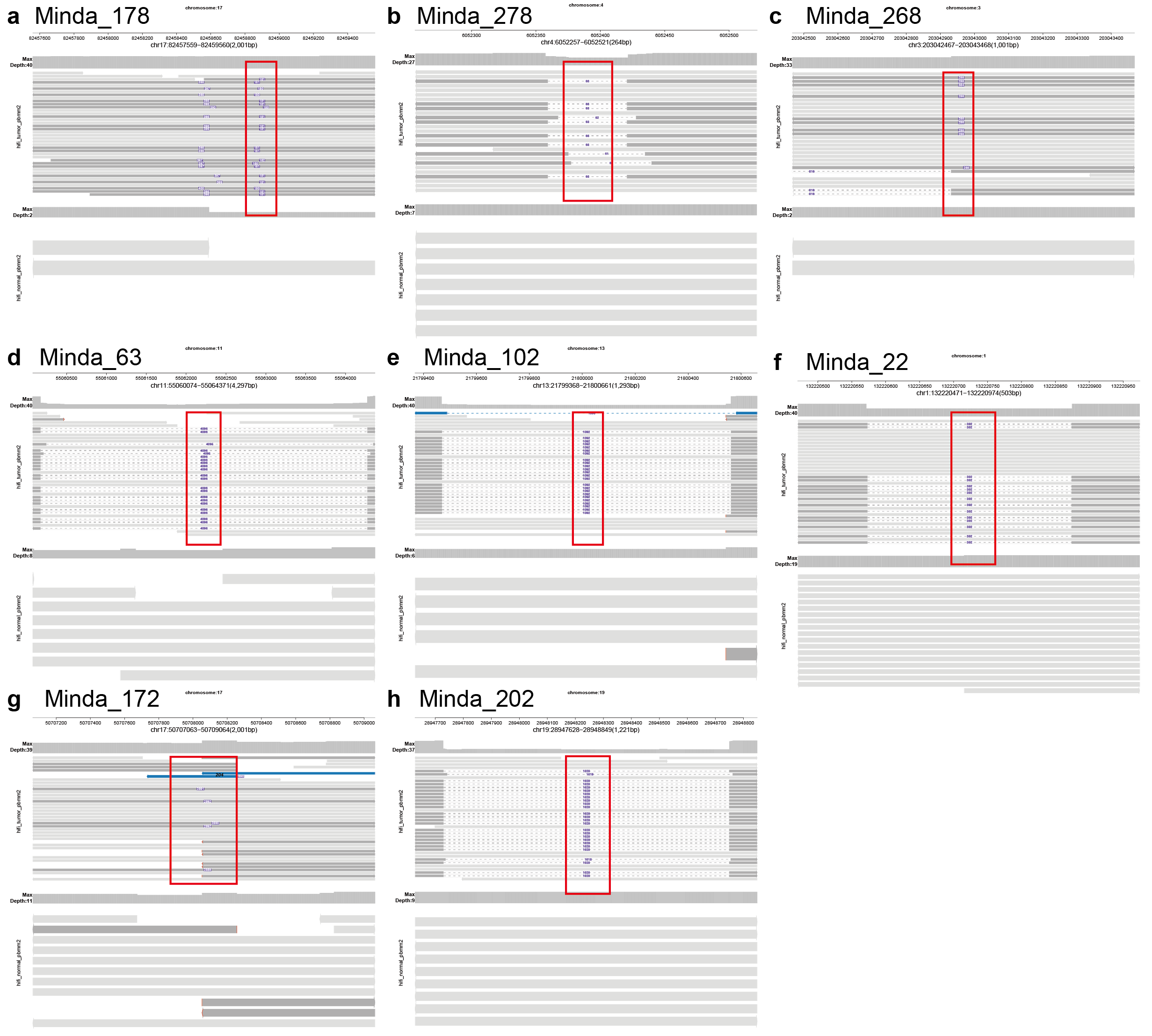


**Supplementary Figure 8. Representative examples of reference-dependent pGenome-unique SVs. a–b,** Examples of pGenome-unique SVs arising from SV type mismatch. **c–d,** pGenome-unique SVs resulting from multi-caller consensus filtering (supporting callers < 3). **e,** Fully mappable breakpoint SV exhibiting cross-chromosomal mapping to GRCh38 or T2T-CHM13. **f,** SVs with breakpoint flanking sequences lacking suitable mapping positions in GRCh38 or T2T-CHM13. **g,** pGenome-unique SV caused by the absence of a TE-rich allele in pGenome relative to GRCh38 and T2T-CHM13. **h,** SV not called in T2T-CHM13 but supported by IGV inspection.


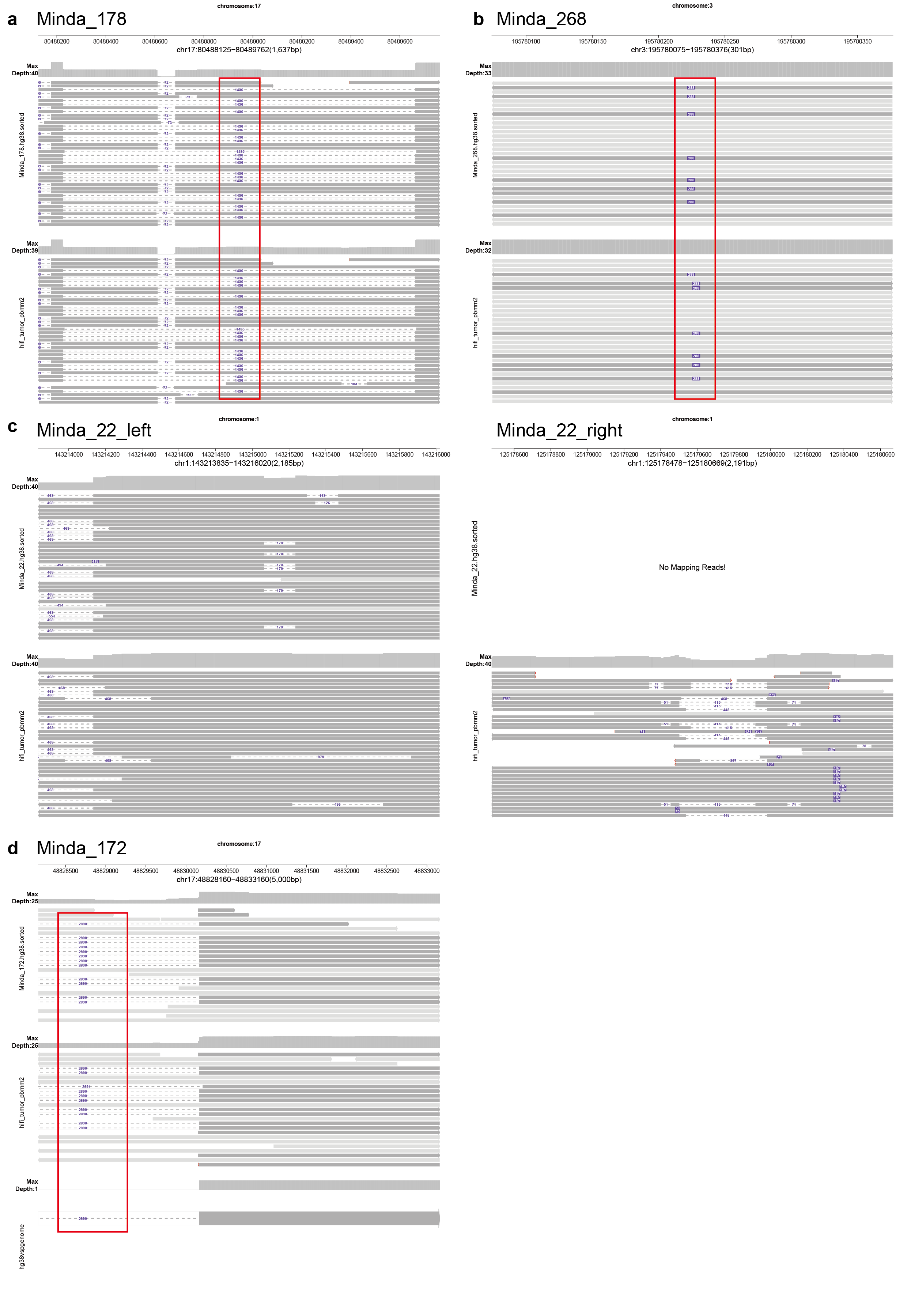


**Supplementary Figure 9. Representative GRCh38-based alignments of pGenome-unique SVs. a,** SV type mismatch example: Minda_178 detected as an INS in pGenome but as a DEL in GRCh38. Top, reads supporting the pGenome-unique SV aligned to GRCh38; bottom, alignment of all tumor HiFi reads. **b,** Caller consensus example in GRCh38, where insufficient caller support prevented SV reporting. **c,** Fully mappable / unmapped breakpoint example (Minda_22), in which ±2000 bp breakpoint flanking sequences map to two distant loci in GRCh38. **d,** Fully mappable breakpoint example (Minda_172), in which the TE-rich sequence is absent from pGenome but incorporated in the GRCh38 reference allele.


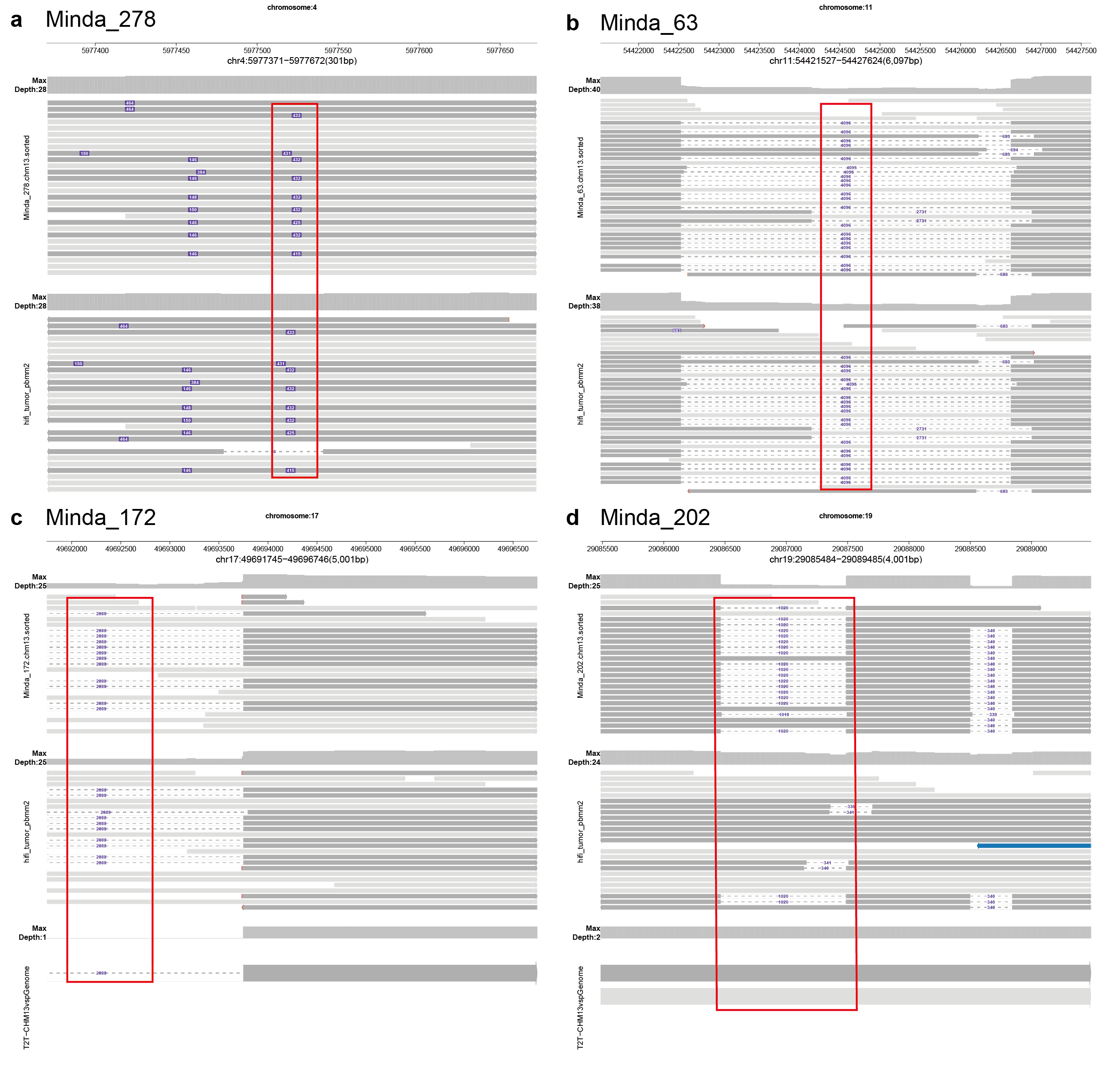


**Supplementary Figure 10. Representative T2T-CHM13-based alignments of pGenome-unique SVs. a,** SV type mismatch example: Minda_278 detected as a DEL in pGenome but as an INS in T2T-CHM13. **b,** Caller consensus example in T2T-CHM13, with insufficient supporting callers. **c,** Fully mappable breakpoint example (Minda_172), in which the TE-rich sequence is absent from pGenome but incorporated in the T2T-CHM13 reference allele. **d,** Unmapped / fully mappable breakpoint example (Minda_202) located in a centromeric region, visible by IGV but not detected by SV callers.


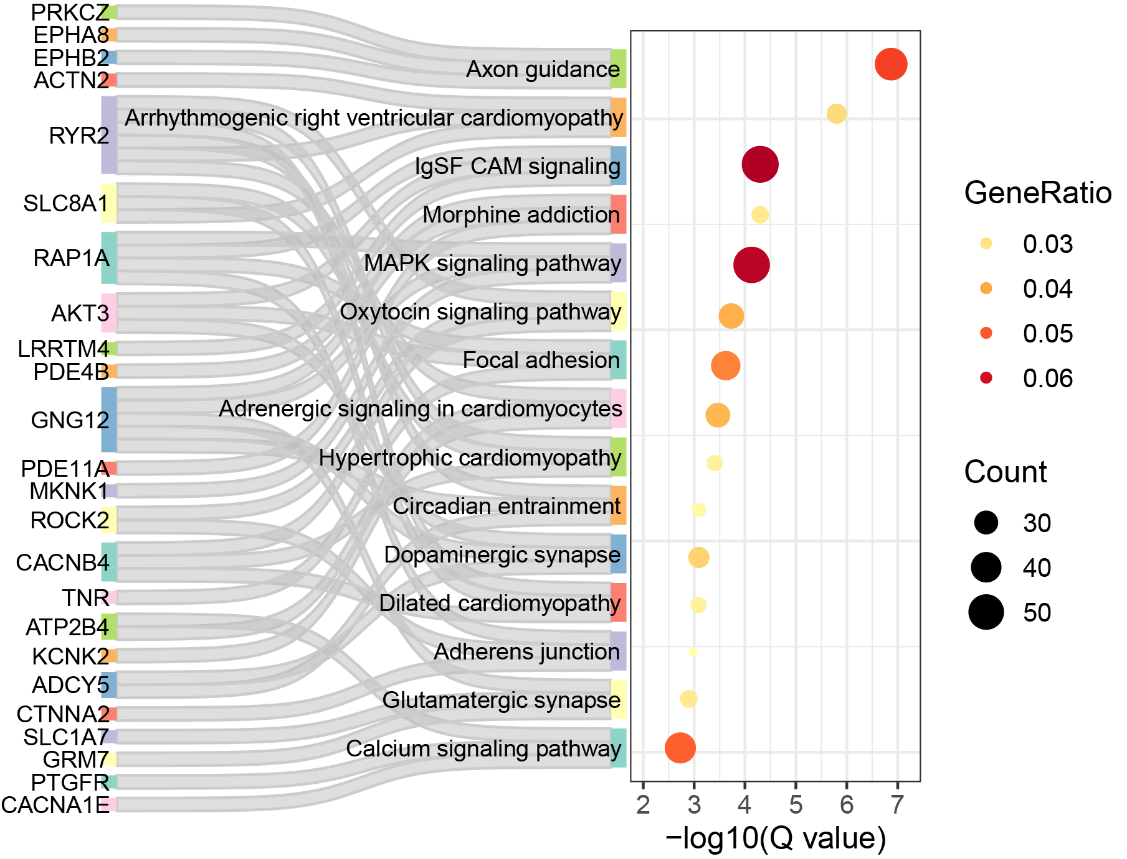


**Supplementary Figure 11.** KEGG pathway enrichment analysis of genes containing SVs that were only detected using the assembly-based method. Pathways were evaluated using Benjamini–Hochberg correction, and retained pathways are shown with FDR values and gene ratios.


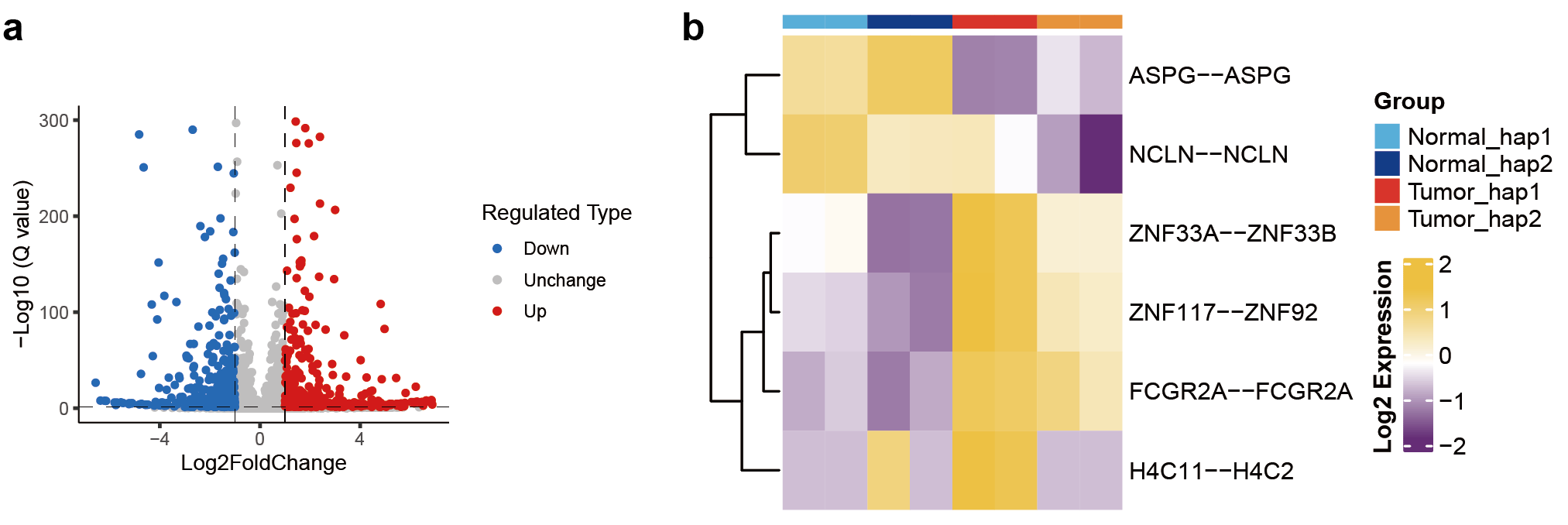


**Supplementary Figure 12. Allele-specific differential gene expression analysis. a**, Volcano plot showing overall differential gene expression between matched tumor and normal RNA-seq samples. Differentially expressed genes were defined by |log2 fold change| > 1 and FDR < 0.05. **b**, Heatmap showing the six dual protein-coding tumor-restricted ASE candidates that were significant in tumor tissue but did not meet the same ASE threshold in matched normal tissue.


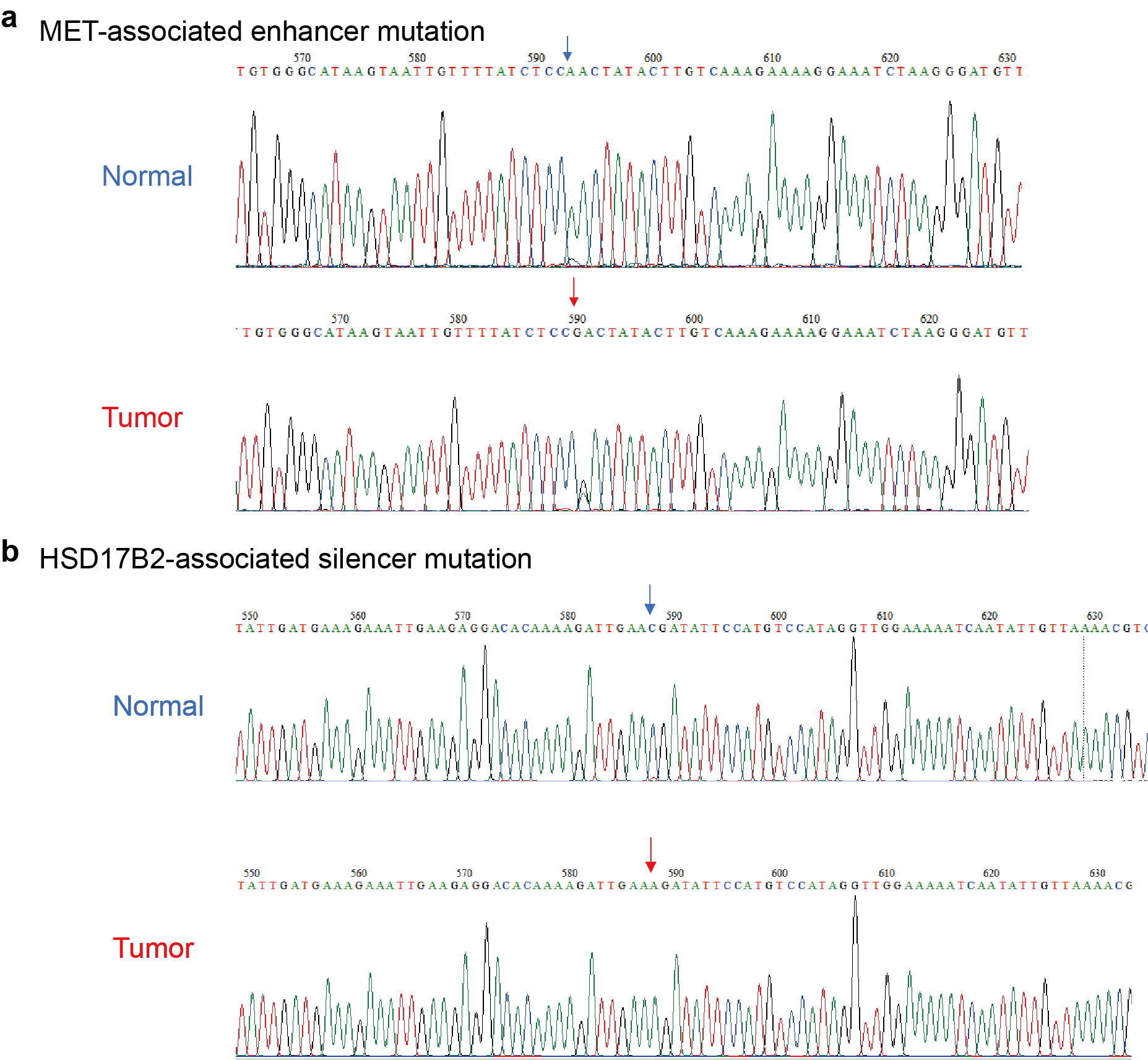


**Supplementary Figure 13. Sanger sequencing validation of candidate regulatory SNVs.**

**a,b,** Sanger chromatograms confirming tumor-specific regulatory SNVs in paired normal and tumor DNA. **a,** MET-associated enhancer mutation. **b,** HSD17B2-associated silencer mutation. Blue arrows indicate the reference alleles in matched normal DNA, and red arrows indicate the corresponding variant alleles in tumor DNA.


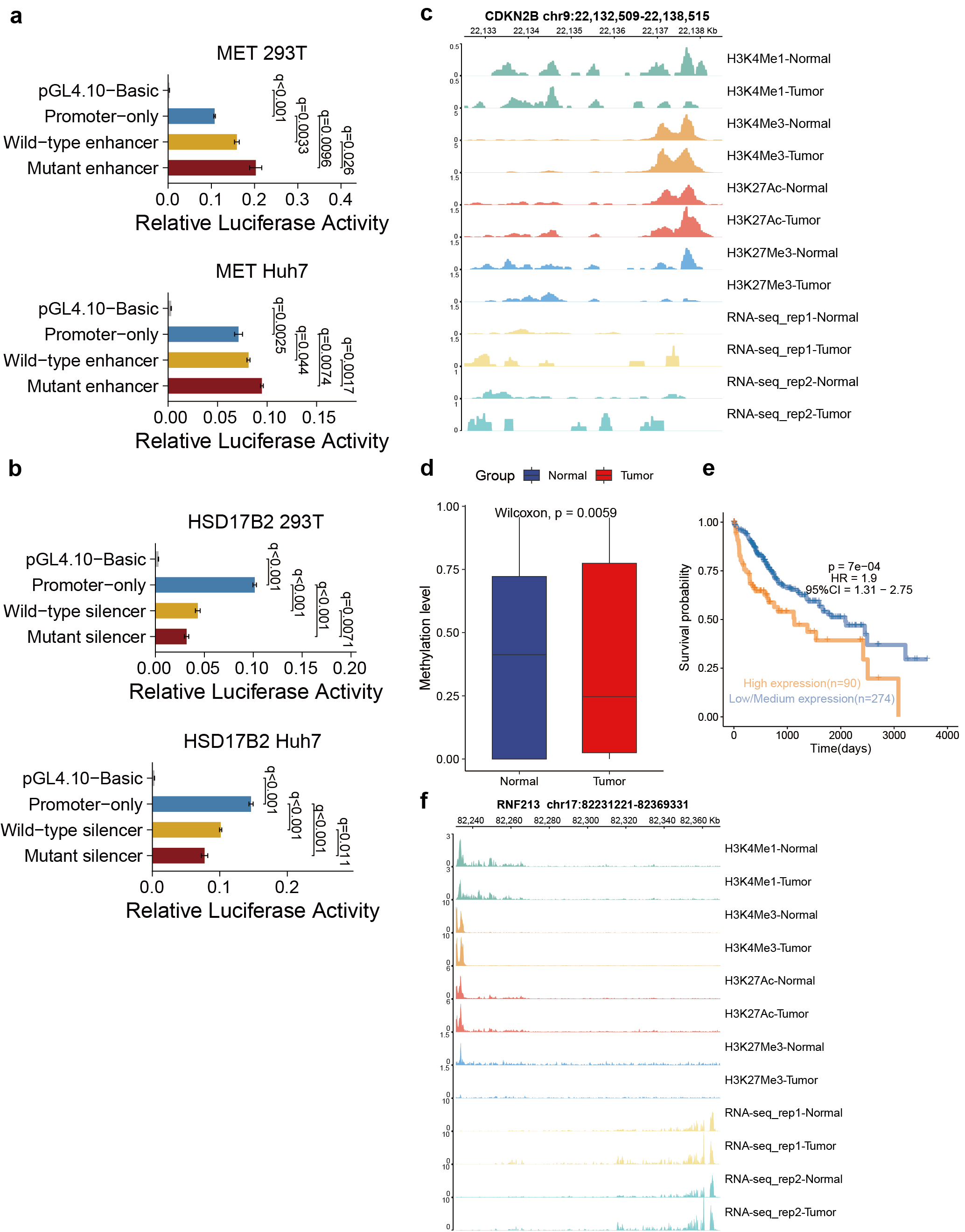


**Supplementary Figure 14. Functional reporter assays and multi-omics profiling of candidate regulatory alterations.** **a**, Luciferase reporter assays of *MET* wild-type and mutant regulatory alleles in 293T and Huh7 cells. **b**, Luciferase reporter assays of *HSD17B2* wild-type and variant-containing regulatory fragments in 293T and Huh7 cells. For all luciferase reporter assays, *Firefly* activity was normalized to *Renilla*, and pairwise Welch-test *P* values were adjusted by Benjamini–Hochberg FDR correction. **c**, Integrative analysis of *CDKN2B* alterations, including histone modification and RNA expression. **d**, *CDKN2B* promoter methylation in normal and tumor samples. **e,** Kaplan–Meier survival analysis of *CDKN2B* expression in the TCGA-LIHC cohort. **f**, Multi-omics profile of *RNF213*, showing histone modification and RNA expression.
